# Abstracts of papers presented at the 1997 Pittsburgh Conference

**DOI:** 10.1155/S1463924697000114

**Published:** 1997


Journal of Automatic Chemistry, Vol. 19, No. 4 (July–August 1997) pp. 91–131

### Abstracts of papers presented at the 1997 Pittsburgh Conference

The following abstracts are of papers presented at this year's Pittcon held in March. The editors have selected, from over 1000 presentations, those of particular interest to the *Journal of Automatic Chemistry's* readers. Pittcon '98 will be held in New Orleans from 1–6 March. For additional information please contact The Pittsburgh Conference, 300 Penn Center Boulevard, Suite 332, Pittsburgh, PA 15235-5503. Tel.: 412 825 3220; fax: 412 825 3224; World Wide Web: http://www.pittcon.org.

### Ladders and fingerprinting: how MALDI-MS broke into the bioanalytical lab

**Paul Knight, Sally U, Chris Sutton and John Cottrell**
*Thermo BioAnalysis Ltd, Hemel Hempstead, Herts HP2 4TG, UK*

It is little more than five years since the first appearance of commercial instruments designed specifically for matrix assisted laser desorption/ionization mass spectrometry (MALDI-MS). Progress has been extremely rapid, with manufacturers now claiming to offer second or third generation technology.

The recent rediscovery of time-lag focusing has enabled linear instruments to achieve mass resolution figures well in excess of 1,000 (fwhm). Thus, the main reason for choosing a reflector based analyser is for post source decay (PSD) studies, such as peptide sequencing. While there is intense interest in PSD sequencing amongst the mass spectrometry community, it is still far from being a routine application.

For protein identification, peptide mass fingerprinting is both rapid and surprisingly specific. For structural information on novel sequences, whether peptide or oligonucleotide, ladder generation using either chemical or enzymatic degradation provides readily interpreted sequence information. Both of these methodologies can be implemented using a good linear TOF system.

A new linear benchtop instrument, Dynamo, was introduced. The design was driven by the requirement to combine good ease of use and high reliability with a performance specification which can address the full range of non-PSD biochemical applications. Mass resolution is 2000 fwhm for peptides, and a rapid sample introduction system allows a 5 cm square target containing up to 100 samples to be introduced and pumped down in less than a minute.

### Lims Validation–a complete life cycle approach

**Siri H. Segalstad**
*Segalstad Consulting, P.O. Box 15 Kjelsås, N-0411 Oslo, Norway*

The reason for validating the system is *not* that FDA or their counterparts in other countries shall be satisfied. It is a good tool for the organization, which will feel more confident that the system performs the way it should—all the time. In order to have a 'validated' system, it must fulfil several requirements. There is no single solution to how to achieve this; there are many solutions, each of which can be equally good. It is fortunate: because you can define what is the best for your organization; and unfortunate: because you have to define it yourself. A rule of thumb is to do what you say and say what you do, and argue in the documents why you do it this way. Among the items which should be included either as separate items or intermingled with each other, are:

- A proper requirements specification must be written before choosing the system. The validation testing later shall prove that the acquired system complies with requirements specification.
- System development must have been done in a quality environment. This can be checked through a vendor audit.
- Installation, qualification must be done according to a pre-planned procedure, and tested according to a pre-planned test.
- Acceptance testing and operation qualification (OQ) may be separate or included in the validation protocol.
- Validation protocol shall be written with a description of the system and the validation process, as well as testplans, error handling and acceptance criteria. All testplans shall include expected result.
- The testing shall be done according to the validation protocol, and documented extensively.
- SOPs for the system must be written. They shall contain security issues, training and access, how to build templates/studies in LIMS, error handling, change control and revalidation issues, daily use of the system, backups, disaster recovery procedures, responsibilities, and other issues which might affect the system.

### Use of surface-enhanced infra-red absorption (SEIRA) spectroscopy for the study of antibody/antigen interaction: a miniaturized biosensor

**Yue Li, Chris. W. Brown and John Seelenbinder**
*Department of Chemistry, University of Rhode Island, Kingston, RI 02881, USA*

In previous work, the authors have demonstrated that surface enhanced FTIR-External Reflectance Spectroscopy (FTIR-ERS) can be used for the characterization

0142-0453/97 $12·00 © 1997 Taylor & Francis Ltd of antibody/antigen interactions. The method is sensitive, fast and specific for each system. The mechanism of the surface enhancement, similar to Surface Enhanced Raman Scattering (SERS), is mainly due to the high electromagnetic field of the surface plasmon polariton (SPP) or collective electron resonances associated with the island nature of the thin metal film. Recently, SERS was observed for near-infra-red (near-IR) excitation (1.06 $\mu$m from a Nd:YAG laser) as strong as for the SERS for visible excitation.

The goal of the present investigation was to explore the feasibility of developing a miniaturized biosensor based on the SEIRA effect. Both near and mid-infra-red spectra of model antibody/antigen systems were measured to determine the optimum regions for selecting analytical wavelengths. Different materials, such as silicon wafers and micro-glass slides, were tested in order to find a low cost substrate. Both gold and silver were coated onto the substrates and their different surface enhancements were studied. In addition to investigating the potential of building miniaturized biosensors, the detection limits, response times, and linearity of quantitation for the sensors have been determined.

### Selection and implementation of a common LIMS in a diverse, global R&D environment

**Martin C. Sammons, James W. Murphy and Donald P. Zimmerman**
*The Procter and Gamble Company, Miami Valley Laboratories, Cincinnati, OH 45253, USA*

Although Procter and Gamble's LIMS experiences go back as far as 1965, success of the various systems has been spotty. Some systems were installed and operated successfully for many years, while other purchased systems, were never even installed. Therefore, Procter and Gamble decided in 1993 to select a common LIMS system for its global R&D Analytical laboratories and to provide an internal support organization for these systems. The challenges were many. LIMS systems would be needed in 21 sites and 12 countries, with vendor support available in at least the majority of the sites. The labs ranged from as few as four to over 100 analytical people with both regulated and unregulated labs. Therefore, the cost structure had to support the budgets of the small labs, while providing performance scalability to reach the needs of the large labs and the configuration flexibility to allow for a wide range of work processes. The LIMS had to fit into the Company's evolving computer infrastructure, which required that the users interface with the LIMS through Windows and placed restrictions on supporting network, server, and database technologies. Each lab needed to be able to configure their LIMS independently, while still allowing for future inter-operability among the systems. And, to make matters more confusing, there was no central analytical management structure making the tough decisions.

This presentation focused on the organizational and technical processes which have been used in LIMS selection and roll-out. Included were the processes of gathering and unifying LIMS requirements from several labs, identifying potential vendors, testing the LIMS products, gaining consensus on the selection, and organizing a roll-out.

### Labware LIMS—SAP/R3 integration in multinational laboratories using QM-IDI

**Jo S. Webber**
*LabWare Inc. Three Mill Rd, Suite 102, Wilmington, DE 19806, USA*

Many corporations are standardizing on the use of SAP R/3 as the Enterprise Resource Planning system (ERP) of choice. SAP does not provide a LIMS module, but instead provides an interface layer and a certification program whereby LIMS suppliers can provide an integrated system link to SAP R/3 and become certified suppliers of complementary products.

In 1996, one of the LabWare's multinational customers embarked on a project to implement an integrated SAP R/3 and LabWare LIMS solution in over fifty of their laboratories worldwide. The project plan required that the LabWare LIMS integration to SAP R/3 appeared seamless to the end users and that the SAP R/3 system was provided with all the same information regardless of whether it originated in LabWare LIMS or was brought in directly into SAP R/3.

This paper discussed the nature of the integrated link between LabWare LIMS and SAP R/3, highlighting the integration points between the two systems. Specific functionality such as the ability to manage skip lot testing in LIMS was addressed and points of overlap between the two systems were examined. A project update on the implementation and rollout of integrated system was provided and the issues that arise when deploying integrated software solutions in multinational laboratories were discussed. Language support issues in SAP R/3 and LabWare LIMS were addressed.

### Development of an HTML based 'Intranet' LIMS data delivery system

**Timothy J. Long**
*Amgen, Inc., One Amgen Center, MS 21-1-C, Thousand Oaks, CA 91320-3847, USA*

One of the major advantages of LIMS is the ability to provide information on the analytical process—creating visibility for analytical information and management of the laboratory. However, with the learning curve on LIMS still fairly steep for those not immersed in laboratory operations, this promise of LIMS' value is diminished.

With the maturing of the LIMS application at Amgen, the need for improved data delivery has become apparent. With the advent of the World Wide Web and HTML(HyperText Markup Language), the capability exists to provide easy access to text based data; however, delivery of relational data through such an interface is still a capability in its infancy.

After analysis of the various alternatives available for data delivery, an HTML based solution was determined to be the best answer based upon issues such as cost, ease of implementation, ease of maintenance, and long term architecture and strategy.

The development process explored such issues as server software and platforms, database access, user interface, what information to deliver, and security. The resultant application is easy to use, available throughout the corporation, and provides a foundation for future growth and enhancement of capabilities.

### The future of analytical instrumentation

**R. Graham Cooks**
*Department of Chemistry, Purdue University, West Lafayette, IN 47907, USA*

Analytical instrumentation and its future was addressed from two perspectives. The first is the straightforward one of where we are now and where extrapolation suggests we are headed. Illustrations from mass spectrometry were used to make the point that the future lies with on-line, real-time, non-destructive, minimally-invasive, multi-dimensional detection and analysis techniques. Minimization of size and maximization of flexibility, portability, applicability to trace (single molecule, half molecule?) analytes in real matrices and, epecially, highly capable data manipulation techniques clearly dominate the present. Data mining of increasingly sophisticated varieties, multi-domain data manipulation and regressively referential data acquisition lie in the future.

It was argued, from a second perspective, that analytical instrumentation represents the heart of modern scientific innovation. Furthermore, the lack of appropriate accommodation of this discipline within the academic establishment constitutes a dissonance which is as much an impediment to scientific progress as is any scarcity of resources. This discipline is peculiarly allied with a scholarship woven around repetitive, commercially-driven and intellectually barren analytical measurements in the subject of analytical chemistry. This alliance has virtues—and these have been made the most of by analytical chemists—but it has major flaws. One consequence is that projects based on the intellectual prowess of the instrumental scientist are seldom directed by members of this group who are too often found in support roles. There are historical and sociological reasons for this state of affairs but not scientific ones. It should not continue.

### Numerical simulation of three-dimensional flows in a discharge tube for helium-microwave induced plasma

**Toshiyuki Suzuki, Hisao Takahara**
*Yokogawa Electric Corporation, 2-9-32 Nakacho, Musashino-shi, Tokyo, 180 Japan*
**and Takatoshii Kasanno**
*Fujitsu Limited, Research Center For Computational Science, 9-3 Nakase 1-Chome, Mihama-Ku, Chiba-Shi, Chiba 261 Japan*

Recently, a particle analyser system (PT1000) was recently developed using helium-microwave induced plasma for direct solid particle analysis. The plasma was formed in a 3.5 mm inner diameter quartz tube. It was stable and had high efficiency to atomize, ionize, and excite most kinds of particles which are in the helium flow. However, it was extremely difficult to directly measure the flow profiles of the helium and the particles in the plasma gas because of the small plasma size and high temperature.

In the authors' previous study, a two-dimensional model was used. This model did not show actual flow patterns inside the plasma confinement tube. Therefore, a three-dimensional numerical model was developed for this study.

Two types of three-dimensional numerical models were used, one is the simplified model which does not include a source of heat and the other is close to the realistic model which includes a source of heat and the adiabatic factors.

This presentation covered the following issues: the calculated condition of three-dimensional numerical models; the pattern of the helium flow; the temperature distribution of the plasma; and the behaviour of these particles in the plasma gas. Further improvements for PT1000 were discussed.

### Reduction of sample introduction errors in graphite furnace atomic absorption spectroscopy

**Dave Pfeil, Gerald Dulude**
*Thermo Jarrell Ash Corporation, 27 Forge Parkway, Franklin, MA 02038, USA*
**and Malcolm Lee**
*Unicam, York Street, Cambridge, CB1 2PX, UK*

Atomic absorption analysis employing graphite furnace atomization has been one of the most sensitive techniques available for the determination of metals, often with detection limits in the parts per trillion range. The technique offers an additional advantage of consuming very small sample quantities, typically 10 μl per determination. Using such small volumes does cause problems in sample transport to the graphite cuvette, which must be recognized and addressed.

Numerous physical problems occur at the sample injector tip. Prior to atomization and analysis of the sample, a mirror inserted into the optimal beam of the hollow cathode lamp after the sample cuvette diverts the beam from the spectrometer detector toward a stationary camera. Photographs of the injector tip in the cuvette during sample deposition demonstrated several of the problems. Analytical results confirmed inaccurate sample deposition. Actions required to overcome deposition errors and results were included.

This presentation showed the effect on results caused by changes to the sample injector position. Efficiency of sample deposition with changing sample volume were shown. The effects of cuvette temperature during deposition, sample surface tension, sample viscosity, and various matrices were discussed.

### Critical evaluation of the determination of lead by flow injection hydride generation atomic absorption spectrometry

**Robert I. Ellis, Julian F. Tyson**
*Department of Chemistry, University of Massachusetts, Box 34510, Amherst, MA 01003-4510, USA*
**Susan McIntosh and Christopher P. Hanna**
*Perkin-Elmer Corporation, 761 Main Avenue, Norwalk, CT 06859-0215, USA*

Flow injection hydride generation (HG) atomic spectrometry is a sensitive and widely developed method for determination of several elements, notably arsenic and selenium. By trapping the hydrides in an electrothermal atomizer, an improvement in sensitivity may be achieved compared with that for direct introduction of the analyte solution into the atomizer. Pre-treatment of the atomizer with trapping reagents has improved the quantitative accumulation of the hydride. The success of these analytical procedures has prompted research into the possibility of determination of other hydride forming elements, including lead.

The authors investigated whether a method based on HG followed by trapping on the interior of a graphite furnace, gives rise to a significant improvement in the detection limit compared with that obtainable by direct introduction of the sample solution into the furnace. In the HG of lead, an oxidizing agent must be present to convert the lead species in solution to Pb(IV) and to prevent reduction to Pb(II) by the borohydride. A number of parameters have been studied including the nature of the oxidant, acid concentration, manifold dimensions, borohydride concentration, argon flow rate, pump delivery rates, furnace trapping temperature and sample volume injected. So far, the best performance has been achieved with 3% ferricyanide in 0.1% HCl solution with 0.5% borohydride, an argon flow rate of 0.15 L/min and trapping on an iridium coating at 300°C. Peak area measurements for a 1,000 µl sample volume gave a detection limit of 0.12 ppb, which is very similar to that obtained for direct introduction of the sample solution.

It appears that although the sensitivity of the HG is considerably greater than that obtained for direct introduction, the precision is significantly poorer. The sources of the imprecision will be determined and the potential for the flow injection HG electrothermal atomic absorption spectrometry technique were discussed.

### An evaluation of the electronic nose profile by multiway principal component analysis

**Su-Chin Lo**
*United States Tobacco Manufacturing Company, Research & Development, 800 Harrison Street, Nashville, TN 37203, USA*

The electronic nose was developed as an aroma monitoring technique that yields classification results for the food and flavour industries. The analysis of aroma is based on two main components which include the chemical sensing elements and the automated pattern recognition system. By selecting the proper sensors and the region(s) of corresponding sampling time, an electronic nose can be applied to specific odour-analysis problems from raw material screening to final product quality evaluation.

In most commercial systems, the preliminary data analysis is based on the selection of a time window with representative sensing patterns and subsequent averaging of the sensor readings over this period of time. The selection of time window is ambiguous and sometimes is a tedious process based on pair-to-pair sample comparison. However, the average of sensor readings does not provide better resolving power if response is complex as a fraction of time. In this study, a multiway principal component analysis (MPCA) was applied to extract total information from the sensor profiles in order to resolve similar aroma patterns. MPCA can be used to explore correlated relationships among samples, sensor responses, and sampling time in three-mode data arrays simultaneously. In addition, the multiway partial least squares (MPLS) shows those sensing variables (sensors and sampling time) which are the main contributors to any differentiation produced. The benefit is allowing one to monitor specific aroma patterns more easily. Results arising from the study of tobacco samples were given.

### Autonomous raw material identification by combining near-infra-red spectroscopy and chemometrics

**Denise E. Root, Bogdan Kurtyka and Timothy G. Kelly**
*Perstorp Analytical, 12101 Tech Road, Silver Spring, MD 20904, USA*

In today's manufacturing environment, the ability to rapidly and unequivocally identify raw materials is of paramount importance. This requirement is necessitated in the pharmaceutical industry by GMP guidelines. In the polymer and chemical industry, raw material identification is used to address ISO requirements, and to ensure product quality and consistency. For post-consumer materials such as carpets and polymers, material identification is used for automated sorting routines.

While near-infra-red (NIR) spectroscopy has long been recognized as a rapid, alternative analytical technique which requires no sample preparation, it is the applied chemometrics which affords the opportunity to perform autonomous raw material identification.

Ideally, one wishes to collect a spectrum and by means of Principal Component Analysis or other Pattern Recognition Routine, isolate the reference spectra to within a specified statistically significant interval. Then, an unknown spectrum is collected, compared to the reference library and identified. In principle this is relatively easy to perform. In practice, however, a variety of materials are spectrally similar and therefore, several libraries are necessary for unequivocal identification. To achieve this requirement, a means of cascading through multiple libraries is necessary. This process can be visualized as a series of branches attached to a limb of a tree. First, we select the appropriate limb and then we isolate the correct branch.

Drawing from applications in the pharmaceutical, polymer, chemical and textile industries, this presentation described the benefits of using cascading libraries for rapid and unequivocal identification of raw materials.

### Supercomputer analysis of high dimensional data sets

**Robert G. Buice, Jr., Kevin D. Donohue and Robert A. Lodder**
*College of Pharmacy, University of Kentucky, Lexington, KY 40536-0082, USA*

Fatal strokes and cerebral ischemia have as a primary cause carotid artery atherosclerotic plaques. Near-IR analysis of carotid plaques has two important uses: it may help us assign appropriate patients to drug therapy instead of surgery. Early plaques with a high lipid content may be treatable with drugs before the patient is made to endure surgery, which is performed when the plaque reaches 60% stenosis. It provides a noninvasive method of studying the mechanism of formation of the plaque *in vivo* to study the role of oxidized LDL in the process.

The inherent problem with the use of near-IR to study biological samples is that the analytes frequently exist at much smaller concentrations that the other constituents of the sample matrix. In biological systems the matrix includes sodium, potassium, chloride, proteins and other components which cause spectral interference and can vary from patient to patient. Various chemometric techniques have been tried to correct this problem, but it is necessary (and simple) to add other noninvasive analytical techniques that will allow us to quantify the error-causing components of the sample matrix and reduce the analytical error in the near-IR determination of biological analytes.

Magnetohydrodynamic Acoustic-Resonance Near-Infrared Spectrometry (MAReNIR) combines three techniques: near-IR, to quantify the analyte; acoustic-resonance spectrometry, which measures the attenuation and velocity of ultrasonic pulses to give information on sample density and bulk properties (such as total protein concentration in biological samples); magnetohydrodynamic spectrometry, to give noninvasive determination of the ionic strength of a sample.

The combination of these three techniques results in a high dimensional data set (usually a matrix of 100 by 12 000 or more) because of the concatenation of spectra from the different techniques. This paper demonstrated how analysis of these data sets (using PCR and BEAST calibration on a Convex Exemplar Supercomputer) leads to a reduction in analytical error over simple near-IR analysis while remaining simple and noninvasive.

### Multivariate analysis of hyperspectral Raman images of complex samples

**Jeremy M. Shaver, Nancy L. Jestel and Michael D. Morris**
*Department of Chemistry, University of Michigan, 930 N. University Ave., Ann Arbor, MI 48109-1055, USA*

Raman imaging of all but the simplest systems requires collection of hyperspectral (many bands, many spectral resolution elements) images for extraction of component or property information distributed throughout the spectra. Good quality hyperspectral image sets can be collected using high-throughput Raman imaging systems which can be filter-based or spectrograph-based. The data sets provide a basis for the study of highly complex samples, such as glasses, minerals or pharmaceutical formulations. Raman imaging studies of various crystalline and glassy materials which contain a variety of molecular moieties and inclusions have indicated that we have reached the limit of standard multivariate approaches, which are essentially extensions of principal components analysis to two dimensions. These techniques have difficulty disentangling broad backgrounds from narrow Raman bands and in dealing with spectra containing multiple bands arising from distinct, but chemically similar components. Unfortunately, these data are too complex to analyse by any other techniques without sacrificing information content. Efforts to improve factor rotation and score determination for factor analysis (FA) suggest that there are several promising routes of analysis which combine systematic factor rotation with minimal operator intervention. Using automated and semi-automated procedures on the hyper-spectral data, consistent results and full signal to noise improvements over univariate analysis can be realized. The exceptionally large spectral data sets also allow for internal checks on eigenvector and factor consistency. This paper demonstrated the utility of these approaches to the analysis of hyperspectral images of heterogeneous silicate glasses.

### Real-time monitoring of particulate and inorganic gases

**Kevin P. Carney, Mike Power, Greg Peters, Oleg Tolstikhine**
*Engineering Division, Argonne National Laboratory – West, P.O. Box 2528, Idaho Falls, ID 83403, USA*
**Jeff Lindner and J. P. Singh**
*Diagnostic Intrumentation and Analysis Laboratory, Mississippi State University, P.O. Drawer MM, Mississippi State, MS 39762, USA*

The Plasma Hearth Process is being developed jointly between Argonne National Laboratory and Science Applications International Corporation to stabilize radioactive waste which contains plutonium, uranium and hazardous constituents such as heavy metals and EPA listed organic compounds. Waste is fed into a high temperature direct current arc, where it is melted and solidified into a glassy slag monolith. Organic material contained in the waste is pyrolysed and swept into a propane based combustion system. The off gas is filtered and passed through an acid gas scrubber prior to being released to the atmosphere.

An FTIR spectrometer, two colour transmissometer, and laser induced breakdown spectrometer system have been installed on the plasma hearth off gas system for on-line, real-time diagnostics. Information obtained from the system include: determining the gas composition of infra-red active molecules such as propane, acetylene, methane, carbon monoxide, carbon dioxide, water, nitric oxide and sulphur dioxide; the determination of the temporal behaviour and elemental composition of particles and particle size distributions; and determining rotational temperatures of the off gas to determine off gas temperatures.

A standard addition manifold has been installed to accurately add known quantities of calibration gases (CO, NO, $SO_2$, $CO_2$ and HCl) to the PHO off gas flow system. Other gases, such as arsine, can be added to the off gas system for metal CEM calibration. Single beam spectra were obtained for CO at concentrations of 315, 630 and 1260 ppm in an off gas flow measured at 225 cfm. A limit of detection of 200 ppm for CO was calculated using the acquired calibration spectra.

Absorbance spectra were obtained during the addition of sulphur dioxide to a gas stream with 140 ppm $SO_2$. The limit of quantification was estimated to be approximately 140 ppm. A linear calibration was obtained for a plot of the integrated absorbance (over a frequency range of 2623 $cm^{-1}$ to 2784 $cm^{-1}$) for a series of calibration standards ranging from 150 ppm to 560 ppm $SO_2$.

A laser induced breakdown spectrometry system has been developed and installed to quantify actinide and EPA listed toxic metals in the off gas system prior to filtration. This information will be used to characterize the efficiency of the thermal treatment system to encapsulate U, Pu and RCRA listed metals for disposal. Calibration and measurement issues for U, Cd, Pb and Pu were briefly discussed.

### On-line monitoring of polyol esterification processes using a microwave based analyser

**Paul R. Geissler**
*Exxon Chemical Company, P.O. Box 241, Baton Rouge LA, 70821, USA*
**Samuel R. Shortes and Bentley N. Scott**
*Phase Dynamics, 1343 Columbia Drive, Richardson TX, 75891, USA*

All materials possess a microwave property which determines the velocity at which a microwave passes through the material and the loss it incurs. This electrical parameter is called the *permittivity* of the material and is related to the molecular dipole moment and relaxation time. Changes in molecular composition, bonding, or structure alter the permittivity of the product stream. By using the effect of permittivity on the output of an unbuffered microwave oscillator, changes in frequency can be calibrated to compositional and even isomeric changes. This phenomenon is called *oscillator load pull* and is the basis of a new measurement technology.

The determination of the extent of esterification of polyols with mixtures of carboxylic acids to produce synlube esters is typically accomplished by time-consuming, complicated, and often impossible-to-do gas chromatographic procedures. This makes the optimization of batch reactor times very difficult. The authors have shown that microwave absorption can be used as a rapid and accurate on-line measure of the conversion of polyols at conversion levels $>95\%$ (equivalent to hydroxyl numbers $<5$), even in the presence of excess acid and ppm levels of water.

The esterification of trimethylol propane with a mixture of linear carboxylic acids was monitored in a pilot plant reactor and the observed frequencies are plotted below as a function of the percentage conversion as determined off-line by gas chromatography.

### Transferring NIR calibrations from the lab to the process: a case study

**Moise Haor**
*LT Industries, Inc., 6110 Executive Blvd., Ste. 800, Rockville, MD 20852, USA*

Near infra-red is a correlative technique. A measurement is performed by 'correlating' the spectra of a sample measured in one analyser with some properties measured by a traditional method. When the analyser changes, the calibration needs to be 'transferred' to the new analyser.

This paper described the steps required to transfer a near infra-red calibration (developed in the lab) to a process analyser, a critical step, as most measurement methods are developed with lab samples off-line. Calibration samples represent a real cost when primary-method analysis, personnel, storage and other costs are involved. The process environment requires a different analyser but the effort invested in the lab calibration can be transferred.

A case study was described, with concrete guidelines for calibration transfer. Comparison of multiple chemometric techniques was presented.

### Fast, automated total fluorine determination in almost any solid sample—from plastics to toothpaste

**Xinwei Yan and K. A. Allan**
*Antek Instruments, Inc., 300 Bammel Westfield Road, Houston, TX 77090, USA*

Fluorine additive levels in polyethylene and other plastics must be carefully maintained to control certain physical and chemical properties, such as their chemical resistance. In certain refinery processes, the accumulation of fluorinated contaminants on alumina based catalysts, acting as poisons, must be monitored to ensure normal production. In consumer goods, fluoride in toothpaste must be measured and maintained below a toxic level. In medical or biological applications, fluorine exposure can be studied by analysing hair or tissue samples. Therefore, a way to measure fluorine concentrations in solids is needed.

Conventional methods for measuring fluorine in these solids are inadequate in some cases and non-existent in others. A few currently used methods have major drawbacks including safety or environmental hazards, being time-consuming and labour-intensive, as well as lacking sensitivity or specificity for fluorine.

The ANTEK alternative, a rapid, sensitive, safe and automated total fluorine determination method based on pyrohydrolysis followed by ion specific electrode (ISE) detection has been developed and successfully applied to many solids applications. In this paper, this new technique is presented, along with applications in many of the above mentioned industries. A very broad linear analytical range is an important aspect of this method. This method has been successfully applied to samples with concentrations ranging from sub parts-per-million to high %F. Many samples have been analysed with precision unequalled by any previous method. The high sensitivity achievable with this method makes it possible to analyse some low level samples which is not otherwise possible. The high specificity for fluorine is another unique feature of this method.

### Automated portable ammonia monitor for sea and waste water

**N. A. Chaniotakis, E. A. Moschou**
*Department of Chemistry, University of Crete, Iraklion 71409, Crete, Greece*
**N. Papandroulakis, P. Divanach**
*Institute of Marine Biology of Crete, P.O. Box 2214 71003 Iraklion, Crete, Greece*
**and S. West**
*ORION Research Inc. 500 Cuymmings Center, Beverly, MA 01915, USA*

The monitoring of ammonia levels in a variety of aqueous environments is an indicative parameter of the quality of the water. Two such systems of great interest are fish-culture environments and the waste waters. Continuous analysers for the monitoring of ammonia are usually based on colorimetric techniques. Even though these methods have the desired sensitivity, they are usually limited to filtered samples due to interferences from suspended matter.

In this presentation, a new, portable, battery-powered flow analysis (FA) system, based on a potentiometric ammonia sensor, was described. The chemistry is optimized for sea water and waste waters. The carrier stream has high capacity for pH buffering and metal ion complexation and the ionic strength of the electrode filling solution is adjusted accordingly. The reaction time is minimized and the reaction coils are constructed in such a way as to overcome any problems from precipitation. The complete system was optimized to operate with unfiltered samples and has a range of detection between 0.05 and 10 ppm with reproducibility of 5% and stability of 0.02 ppm/hr at constant temperature, while the analysis time is of the order of 1 to 3 minutes, depending on the sample analysed.

Recovery studies as well as the comparison with the standard colorimetric method prove the suitability of the instrument for precise analyses of ammonia for both lab and field applications.

### Design, operation and performance of a digital signal processor spectrometer

**Rick Hapanowicz**
*Nicolet Instruments, 5225-5 Verona Rd., Madison, WI 53711, USA*

Digital signal processing (DSP) technology is rapidly becoming a key part of FT-IR instrumentation and has dramatically improved the quality and availability of advanced step-scan and time resolved experimentation. DSP reduces the level of electronics required external to the main instrument and, coupled with improved user interfaces, brings these complicated experiments into the realm of the normal research laboratory. This work described the design and theory of operation of a DSP based FT-IR. The instrument configuration used for these experiments and the advantages of using built-in digital signal processing for linear-scanning, step-scanning, the collection of multiple data channels and time-resolved spectroscopy techniques were discussed.

### New analytical developments in a combustion analyser for the automatic routine determination of nitrogen in lubricants

**Liliana Krotz, Luigi Ragaglia and Franca Andreolini**
*CE Instruments, Strada Rivoltana, 20090 Rodano (MI), Italy*

In a typical production process of mineral oils, the nitrogen content is periodically monitored and tested for quality control. Therefore the day-to-day reproducibility is an important parameter. Analytical methods employed and the instrumentation used for this purpose have to be proven very reliable and able to cope with all the requirements typical of a routine quality control type of analysis.

The analyser utilized for the work described operates according to the dynamic flash combustion method. This method provides a quantitative and instantaneous combustion of the lubricant sample, completely converting the nitrogen present in the sample, regardless of its chemical structure, into its elemental form. The nitrogen gas is separated by the other combustion gases formed by means of adsorption filters and a GC column, finally it is detected by a thermal conductivity detector.

Improvements in the carrier gas control through an electronic device have been implemented in the instrument used in order to achieve an extremely elevated stability of analytical conditions, allowing the analysis of a large number of samples without the need of recalibration.

The use of a new original-design stainless steel combustion tube housing a special crucible that makes ash removal easier, and the optimum combustion conditions achieved with the oxygen-enriched atmosphere allow the sample weight to be increased.

This paper presented results on the accuracy and day-to-day reproducibility of the nitrogen determination in lubricants and lubricant additives following QC requirements.

### Automated SPC reporting from X-ray sedimentation particle size distribution analysis

**Anthony Thornton**
*Micromeritics, One Micromeritics Drive, Norcross, GA 30093, USA*

The use of Statistical Process Control (SPC) for monitoring material reliability continues to grow at an accelerated pace. Manufacturing processes and incoming material receipts both include steps where material statistics are evaluated versus reference specifications and/or past history. Graphic representation of these comparisons eases verification of compliance. Use of such graphs is often dictated by an organization's documented Quality Process Procedures. Automated production of these representations and the statistical base for them, where data are taken directly from the instrumental analysis of the material, would greatly assist production and quality managers to validate their materials.

The SediGraph 5100 has long been used to control production and quality of many powdered materials. X-ray sedimentation has been the prescribed method for particle size determination for many industries. A new software package for the SediGraph now includes calculation of SPC parameters, production and printing of control charts, and capability to perform regression analysis between measured size and entered process parameters. These new capabilities eliminate the need for off-instrument post-analysis SPC calculation and reporting. With new networking capabilities, quality statistics can be delivered directly to process and quality management, as well as to the final customer.

### Data prepocessing method for enhancement of two-dimensional correlation analysis

**Angela D. Serra and Hugh H. Richardson**
*Department of Chemistry, Clippinger Laboratories, Ohio University, Athens, OH 45701, USA*

A number of factors influence the resolution of the synchronous and asynchronous two-dimensional (2D) correlation spectra. For example the authors' analysis shows that the asynchronous spectrum is extremely sensitive to noise, while the synchronous spectrum is sensitive to the spectral resolution of the data.

A simple, three step method to preprocess data for better resolution in 2D correlation was introduced. Interpolation is the first of these steps required to enhance the peaks in the 2D correlation spectra. After interpolation, noise is suppressed from the spectra using single-value decomposition (SVD). The peaks in the synchronous correlation spectrum are resolved by taking the second derivative of the original data. This preprocessing method is discussed in detail.

Twenty-five data points were used to simulate four Lorenzian peaks that decay with time. Two peaks placed at 1110 and 1135 $cm^{-1}$ decay at the same rate, while the other two peaks at 1112.5 and 1137.5 $cm^{-1}$ decay at a slower rate. The simulated data have a signal to noise ratio of 20. The data were preprocessed using the proposed method of interpolation, SVD, and second derivative. After this transformation, 2D correlation resolved all four peaks in both the synchronous and asynchronous spectra. More detailed examples of 2D correlation spectra using the preprocessing method on simulated data were presented.

### Chemical monitoring of surface-enhanced Raman spectroscopy at a silver electrode

**Shi-Ring Chen**
*Chemistry Department, Howard University, Washington, DC 25009, USA*
**Neil Pothier**
*Ceimic Corp., 10 Dean Knauss Drive, Narragansett, RI 02882, USA*
**R. Ken Force and James L. F. Fasching**
*Chemistry Department, University of Rhode Island, Kingston, RI 02881, USA*

Surface-enhanced Raman spectroscopy (SERS) at a silver electrode has been applied in the chemical analysis of compounds of interest. This vibrational spectroscopic monitoring technique can be incorporated for analytes adsorbed at a silver electrode. The objective is to connect a low-volume SERS detector cell to a flowing stream under dynamic conditions and collect the vibrational fingerprint spectra of biologically important compounds. An argon ion laser was used as an incident excitation and focused on a working electrode by a double convex lens. The scattered SERS signals were collected and focused by a Rexatar camera lens and the incident excitation was attenuated by a holographic edge filter. A multichannel Raman spectroscopy contains a charge-coupled device (CCD) detector and a single spectrograph. This SERS technique has been interfaced with a flow injection analysis (FIA) system to detect selected aromatic amino acids and dipeptides at a silver electrode surface. The applied electropotential study of analytes has indicated the potential where the largest SERS enhancement of compounds occurred. SER spectra of analytes can be reproducibly collected in a carbonate buffer under dynamic conditions. The molecules are able to be efficiently adsorbed and desorbed at the silver electrode surface by appropriate electrochemical modulation. This SERS system is capable of simultaneously monitoring two compounds under flowing conditions. These experimental results demonstrate that SERS performed at a silver electrode can obtain vibrational information and qualitative structural identification of molecules eluting from a flowing stream.

### Re-engineering the analytical laboratory

**Anthony J. Montana**
*Revlon Research Center, Inc., Analytical R&D, Edison, NJ 08818, USA*

Re-engineering is defined as the fundamental rethinking and redesign of an organization's basic processes to achieve dramatic improvements in critical measures of performance such as cost, quality, service, and time. With downsizings and reorganizations commonplace within industry today, many analytical laboratories face a tremendous challenge in maintaining peak operational efficiency during periods of staff and resource constraints. Though many laboratory re-engineering initiatives have been reported, they frequently have not proven to be successful, and they often get lost in the flow of other cost-cutting objectives within an organization. This presentation provided recommendations for the successful start-up and implementation of a re-engineering process under less than perfect conditions.

### Application of multivariate calibration methods to radio frequency glow discharge atomic emission spectrometry rf-GD-AES

**Yuancai Ye and R. Kenneth Marcus**
*Department of Chemistry, Howard L. Hunter Laboratories, Clemson University, Clemson, SC 29634-1905, USA*

The precision and accuracy of measurements are important figures of merit which have to be considered by analysts. In addition to the optimization of instrumentation, the application of various multivariate calibration methods, such as multiline regression (MLR), principal component regression (PCR), and partial least squares regression (PLS) methods, can be an effective way to improve the precision and accuracy of analytical results. For over 10 years, the above three calibration methods have been successfully applied particularly to analysing and modelling the experimental data of molecular band spectra. However, these methods are rarely used to regress the data of line spectra. This is probably due to the fact that it is difficult to find a large enough number of spectral lines with good S/N values for some elements.

The main goal the study described was to improve the precision and accuracy of rf-GD-AES measurements. The measurement of six elements in a series of low alloy steels was successfully made by rf-GD-AES, in combination with two popular modern multivariate calibration methods: PCR and PLS. The results obtained indicated that the application of various multivariate calibration methods to rf-GD-AES is a very promising way to improve effectively the measurement precision and accuracy. The %RSD values can be reduced from about 6% down to around 2% for the analysis of steel samples. Both PCR and PLS methods work considerably better than conventional internal reference methods. The reconstruction of special data demonstrates that this is a feasible and simple way to solve the problem of limited availability of spectral lines with good S/N values in atomic emission spectrometries using Fe matrix spectral lines. Because PCR and PLS are basically statistical methods, the more data and more training standard samples used, the more accurate the model result. Normally more than a dozen calibration samples are needed for the application of PCR and PLS methods. Unlike solution techniques, there is little chance to have this many standard samples for the analysis of solid samples. To solve this problem, reconstructed spectra were modelled for each element in the study. The results showed that it would be possible to use four or more standard samples with three or more replicates of each sample for the application of PCR and PLS methods.

### Application of experimental design to generate relevant information and representative calibration data

**Suzanne Schönkopf and Dominique Guyot**

*Camo AS, Olav Tryggvasonsgt. 24, N-7011 Trondheim, Norway*

The basic requirement for all modelling is representative data. The major objectives of statistical experimental design is to ensure that we collect data that contain relevant information and to do this in an economical way. Relevant information requires collection of representative samples that span all important variations.

The principles of experimental design have traditionally been used to plan efficient experiments, but is equally useful to collect good calibration data, to speed up method development, and to improve process and quality analysis.

Several approaches were illustrated in this presentation through a range of application examples. Data can be selected from available samples or produce samples according to an experimental design plan with the intention to span all important variations. The authors explained the concept of factorial designs, and presented two ways of applying experimental design to spectroscopy calibration; either to focus on some of the parameters of interest directly (reference values), or on the underlying conditions which generate consistent variations in the spectra, e.g. production factors. A third alternative is to use the philosophy of experimental design to select samples from an existing population.

The concept should be used to select both calibration samples and validation samples.

### Making a smart instrument

**Paul J. Rauch, Peter de B. Harrington**
*Center for Intelligent Chemical Instrumentation, Clippinger Laboratories, Chemistry Department, Athens, OH 45701-2979, USA*
**and Dennis M. Davis**
*US Army, Aberdeen Proving Grounds, MD 21010-5432, USA*

When the concentrations of mixture components vary independently with time, pure components may be resolved using chemometric methods such as SIMPLISMA. SIMPLISMA has proved useful for resolving analyte features of ion mobility spectra acquired from vapour phase mixtures. When sampling vapours obtained from liquid mixtures, the analyte vapour compositions may not vary independently. For ion mobility spectrometers (IMS), a vapour mixture may be resolved by differential clear down rates. Clear down is the decay in analyte signal when the instrument is removed from the sample. For ion mobility spectrometers the sample is removed by adsorption onto molecular sieves inside the instrument. If different mixture components exhibit different affinities for the molecular sieves or different vapour pressures, the concentration of the mixture components will vary independently during clear down and SIMPLISMA may resolve the components. This approach is an example of reverse frontal chromatography for which the sample is introduced into the detector (the iMS) and removed chromatographically by adsorption onto the molecular sieves.

In this presentation three chemical agent simulants; dimethyl methylphosponate (DMMP), diethyl methylphosphonate (DEMP), and diisopropyl methylphosphonate (DIMP); were used to demonstrate this new data extraction method with SIMPLISMA. The IMS used was a Chemical Agent Monitor (CAM) Type 482-301N

(Grasby Ionics, Ltd. Watford, Herts, UK) and was employed with only a single modification. The reagent chemistry was based on water, rather than acetone, and this was attained by removing the reagent gas source from the recirculated gas system.

### Efficiency of the heart-cutting process

**Matthew Peters and Joe M. Davis**
*Department of Chemistry and Biochemistry, Southern Illinois University at Carbondale, Carbondale, IL 62901-4409, USA*

Statistical models of overlap have been used to assess the extent of overlap in one- and multi-dimensional separations. Although multi-dimensional separations are powerful means for addressing overlap, they are not widely used. In contrast, chromatographic heart-cutting, in which a small volume of effluent is passed from one column to another for further separation, is used commonly to deal with problems resulting from peak overlap.

Here, probability theory is presented that describes the efficiency of the heart-cutting process for a specific case. The separation on the first column is modelled by homogeneous Poisson statistics, in which the number of components in a heart-cut of temporal width $w$ is unknown. However, the saturation $\alpha$ of the separation is known or can be determined. The separation on the second column is modelled by uniformly random variables, in which the number of transferred components is partially composed of the components whose zone centres lay in the heart-cut. Also transferred to the second column, however, are components whose centres were within roughly one peak's width of the heart-cut boundaries; diffusion and other dispersion mechanisms transport these zones into the heart-cut.

By using Bayes' theorem, one then can calculate the probability that $m$ components were subject to heart-cutting, given the number $p'$ of peaks observed on the first column, the width $w$ of the heart-cut on the first column, the peak capacity $n_c'$ of the heart-cut on the first column, the saturation $\alpha$ of the separation on the first column, the number $p$ of peaks observed on the second column, and the peak capacity $n_c$ of the entire second column. Theory agrees quite well with simple computer simulations, in which the components of the heart-cut are represented by points located at the centre of single-component peaks. More detailed simulations that mimic actual peaks in separations currently are under development to test theory further.

### Client/server computing advances laboratory operations

**Boyd D. Kirkland**
*Hewlett-Packard Co., Palo Alto, CA 94304, USA*

Traditionally laboratory managers have met the challenge of reducing cost, while increasing productivity, by shortening analysis time. That is no longer the solution. Improving results management and simplifying sample preparation now provide the largest potential for improvement. Client/server computing enables the change. Client/server computing is the distribution of task across multiple computers, each with unique capabilities, connected via a network. Clients request actions or information and servers respond to those requests. A computer may be a client when it requests information and a server when it responds to a request from another system. While, at first glance, this may appear complex, the flexibility is a linchpin of client/server computing's power. Each system does the job it performs best, whether it is working as a client or as a server.

An example of how client-server computing could be employed is the distribution of computing and user interaction tasks. Microsoft Windows is accepted by users, many individuals are familiar with its operation, and PCs are ubiquitous. However, desktop PCs do not make idea servers because they are not well suited for large-scale computation or extensive data acquisition.

Within this example, PC-based clients provide users a Microsoft Windows-based interface to enter data, setup instruments, monitor their operation, and enter other instructions. In turn, the server uses this information to acquire data from multiple instruments, simultaneously perform extensive computations on those data, as well as produce reports, and providing long-term, secure data storage. Laboratories can experience productivity gains as well as enhanced regulatory compliance from this type of client/server design.

The client/server design could be extended so the computation tasks are distributed. The clients assume the additional responsibility of allowing users to perform simple, first-pass data analysis and review on individual tests for which they are responsible. The server continues to acquire data, and to perform extensive analysis on data from multiple instruments.

### Classification and bulk characterization of edible oils by discriminant and PLS analysis employing mid-IR fibre optic sampling

**Ashraf A. Ismail, Alham Kermasha, Jacqueline Sedman, F. R. van de Voort**
*McGill IR Group, Macdonald Campus of McGill University, Ste. Anne de Bellevue, Québec, Canada H9X 3V9*
**Mark Druy**
*Sensiv Ltd., Waltham, MA 02154, USA*
**and Cindy Baulsir**
*Spectra-Tech Inc., Shelton, CT 06484-0869, USA*

The edible oils utilized by the food industry are obtained from a wide variety of plant sources. Regardless of their origin, these oils are composed predominantly (>95%) of triglycerides (triesters of fatty acids and glycerol), but the triglyceride makeup (i.e., fatty acid composition and distribution on the glycerol backbone) differs among oils from different plant sources. These differences give rise to variations in the physicochemical, functional, and nutritional properties that govern the suitability of oils for particular food uses. Accordingly, the availability of a rapid screening technique for classification and bulk characterization of incoming oils would be of substantial benefit to the food industry.

The implementation of FTIR methodologies that have been developed for edible oil analysis, coupled with the use of FTIR fibre-optic probes, can potentially provide such a rapid screening technique. Accordingly, in the present work, the applicability of mid-IR fibre optics in the classification and bulk characterization of edible oils was evaluated. The attenuated total reflectance (ATR) spectra of commercially available vegetable oils and oil blends were recorded with a fibre-optic probe and employed to develop an oil classification method based on discriminant analysis.

The spectra acquired with the fibre-optic probe were also employed for the determination of the iodine value (IV, a measure of total unsaturation) of the oils, using PLS, and the IV predictions were compared to those obtained from the spectra of the same samples recorded in a 25-μm transmission cell. The practical advantages and limitations of fibre-optic sampling techniques for edible oil analysis applications were discussed.

### New technology in PC based laboratory information management systems (LIMS)

**Donald J. Kolva**
*Accelerated Technology Laboratories, 940 Emmett Ave., Suite 12, Belmont, CA 94002, USA*

A laboratory implementing a LIMS has two choices of LIMS, a small Personal Computer (PC) based on a large UNIX based system. The PC based LIMS works well in small to mid-size organizations and the UNIX based systems work well in large organizations. problems arise when the information has to be shared between the organizations. This can occur when a 'departmental' PC based LIMS shares information with a company wide UNIX based LIMS or when a satellite laboratory shares information with a larger central laboratory. It would be ideal if the same LIMS could operate in both locations.

Fortunately, the differences between PCs and mini computers is diminishing. Windows or Windows NT can be found on both platforms and this can be used to leverage the similarities between the LIMS systems. Imagine a system where the small laboratory is using a PC based system and the large laboratory is using the same system in a client/server configuration connected to a SQL database. Sharing information between the laboratories is accomplished without further adaptation because both laboratories are running the same software. If the software could provide access from the Internet or a company Intranet, the same system could be used throughout the organization.

This presentation outlined the advantages of a local and client/server Windows LIMS over the older UNIX based systems. These advantages include scalability, familiarity with the Windows operating systems by the laboratory personnel, variety of available software, lower cost, importing and exporting of data to other applications and, most important of all, ease of use.

The author discussed features that today's competitive laboratories demand, such as sample tracking, bar coding, automatic reporting, instrument interfacing, etc. The typical PC Local Area Network (LAN) configuration for laboratories and selection strategies for LIMS were also discussed.

### LIMS assisted decisions: quality at the bench

**Lisa V. Milenkovic**
*Quality Automation Sciences, Inc., 750 Verona Lake Dr., Weston, FL 33326, USA*
**and Mary T. Curtin**
*Massachusetts Water Resources Authority, Boston, MA 02129, USA*

Environmental laboratories are required to maintain strict quality standards capable of withstanding legal scrutiny. The first line of quality within the laboratory resides with the bench level technician performing the analysis. This talk discussed recent advances in using LIMS to assist the chemist in decisions regarding the quality of their data. This new quality control system calculates all quality control parameters and prompts the analyst for immediate decisions regarding the disposition of the samples. The analyst has several tools available to assist in these decisions, incuding 'hot-key' control charts and automatic chemist training verification. The LIMS also uses links to the solutions and instrument maintenance systems in order to verify that the lot numbers of standards used are valid and non-expired and the instrument is properly calibrated and maintained. Through the automation of the data review steps at the analyst level, the environmental laboratory is able to improve data quality and shorten turn around time.

### GLP compliance simplified with state-of-the-art chromatography data systems

**Lisa A. Fay, Soheil Saadat and Larry Robison**
*Scientific Software, Inc., 3000 Executive Parkway, San Ramon, CA 94583, USA*

Recent advancements in software technology have made it possible to vastly improve the GLP compliance available directly from a new chromatography data system using the Windows NT/95 environment. Each of six compliance categories were discussed, along with the changes and improvements now available in new, 32-bit, advanced chromatography data systems for each of these compliance categories. They included the following.

Advanced automated audit trails, and the accompanying detailed log files, are a significant advancement for GLP system compliance. Every change in the system can be recorded, and the user has the opportunity to note the reason for each change, which also will appear in the log file. These changes can occur in the method, integration, system configuration, system administration, ANY portion of the system. This log file is now saved with each data file, and readily available for future audits.

System protection can include multi-level password protection, file protection, and overall system security. Multi-level password protection can secure small items like files and methods, or more global items, like the instrument, or any method changes from the technician level employee. In addition to this password protection, files can be protected from any overwriting, or duplicates. Overall system security can be achieved with separate log-on passwords for each workstation, user, and instrument.

GLP compliance can be further assisted through the use of interactive, multi-pass system suitability. System suitability involves testing the chromatography unit, or system, and assuring passivity before starting a series of analyses. This suitability can be updated, or checked in the middle of the analysis series. If the system fails to meet any of the preset criteria, the system is designated 'unsuitable', and there are a limited number of alternatives available to salvage the results. This salvage is performed easier, and unattended, with interactive system suitability software. The system can be set to reinject any number of samples, followed by a recalculation of suitability, run an alternative sample in the sequence, continue, or run a shutdown assay. With this intelligence built-in to the system, suitability compliance is simplified, and automated.

### Interfacing laboratory automation systems with SAP R/3

**Herbert Knoesel, Andrew Grygiel and Mike Rank**
*Hewlett Packard, Chemical Analysis Solutions Division, 1601 California Ave, Palo Alto, CA 94304, USA*

More and more companies worldwide are implementing integrated business application solutions to manage all functions essential to their operations. Integrated Information Management (IIM) delivers a competitive advantage by streamlining processes within the companies. To achieve complete enterprise-wide IIM, the laboratory operation has to be integrated with the enterprise as well. Benefits of IIM are:

- Enterprise-wide visibility of laboratory data.
- Reduced complexity through 'plug & play' modules.
- Makes infrastructure as flexible as possible.
- Delivers 'integrated platform' for complete solution.

The leading global provider in business application solutions is SAP, and the R/3 suite of products is accepted as the standard in key industries. This paper discussed how a Laboratory Management System (LIMS) and the SAP R/3 Quality Module complement each other to achieve a streamlined process.

### Determining the efficacy of a production environment by computer simulations

**Eriks E. Smidchens**
*Specialty Gas Department, Great Lakes Airgas, 1250 W, Washington St., West Chicago, IL 60185, USA*

To produce something systematically, there is a dependence on some sort of methodology. However, errors may occur. Therefore, the methodology employed should be able to cope with these problems. Ideally, two things should happen when an error arises. The existence of the error becomes known, and secondly, the source of the error becomes known.

In the production environment under consideration source material $X$ is used to produce mixtures of various concentrations. The concentrations are tested against independent standards containing substance $X$. In the simplified model used to simulate this production environment, the only sources of error are the source material and the standard. An error is considered to be a violation of some guaranteed specification. If the supplier of the standard guarantees the composition to be $1.00 \pm 0.010\%$ material $X$, but in actuality the concentration is 1.016%, then an error has occurred. Similarly, if the supplier of the source material guarantees that the purity is greater than 99.0%, but in actuality the source material is only 98.2% pure, then an error has occurred. If an error occurs because the allowable limit is exceeded by a small amount, or several errors occur simultaneously an obvious identification of the problem's source is obscured creating a need for an in-depth statistical analysis.

Various probability distributions are assigned to each step in the production process. Computer simulations generated by programs developed with C++ and Matlab create independent mixtures that are to be tested against the standard. The results are processed by statistical means to draw an inference about the existence and source of an error. It is of interest to investigate how well the statistical tests employed in the methodology identify the various types of errors.

### Use of near infra-red analysers in real time monitoring of gasoline blending

**Al Annamalai**
*UOP, Guided Wave Process Analytical Systems, 5190 Golden Foothill Parkway, El Dorado Hills, CA 95762, USA*

Gasoline refineries face the challenge of keeping the product parameters within specified ranges to meet the performance, environmental and economic requirements. Feed-stocks used in gasoline production of a typical refinery vary from time to time for various reasons including the different sources of the crude oil. Often, the commercial specifications of a gasoline product are met only after re-blending the original blends following a laboratory analysis. Re-blending increases the production cost.

By monitoring the gasoline parameters continuously during the blending process and using a feed-back control, blends that meet the required specifications can be made the first time, without having a need for re-blending. Near infra-red (NIR) on-line analysers provide a convenient way of measuring several critical parameters simultaneously and quickly in a gasoline blender. NIR analysers also have helped to identify some problems in the refinery process. Without these analysers, the problems could have gone unnoticed for a long-time.

To monitor the gasoline parameters using NIR, calibration models must be developed for these parameters. Different parameters pose different levels of calibration challenges. Currently, we are using NIR analysers to monitor octane numbers, total aromatics, total olefins, total oxygenates, benzene, distillation points, Reid vapour pressure, etc. The authors presented on-line data for these parameters and discussed calibration strategies that were necessary to reach good performance results.

### Validation and use of a calibration for total % hydrogen and index of refraction in hydrocarbons on several FT-NIR analysers without any correction for spectral response

**Henry Buijs, Christine Simard**
*Bomem Inc., 450 St-Jean-Baptiste Ave, Québec, Canada G2E 5S5*
**Uwe Hoffmann, Laurence Parpot and Nathalie Zanier**
*Institut français due pétrole, France*

It is now possible to produce an FT-NIR which incorporates design features and manufacturing procedures such that each unit reproduces the same spectral response consistently over a long time and from unit to unit with a high degree of reproducibility. The reproducibility approaches the repeatability for which FT-NIR is well known. With such an FT-NIR it is possible to develop analysis methods that do not drift with time and that can be transferred from analyser to analyser without any correction for spectral response. This concept has been tested on several analysers and over a period of more than one year for the analysis of total % hydrogen and index of refraction in a class of hydrocarbons.

This paper included details of the implemented analysis method, analysis performance and validation procedures. The latter includes system validation at time of manufacture using a standard sample, regular system checks which give assurance that the system is in specification and check sample validation.

### Identification and qualification of lubricant additives by NIR spectroscopy

**Donald Muller**
*Bran + Luebbe Inc., 1025 Busch Parkway, Buffalo Grove, IL 60089, USA*
**Dagmar Timm, Uwe Grummisch and Horst Grunewald**
*Bran + Luebbe GmbH, Werkstrasse 4, D-22844 Norderstedt, Germany*

Near infra-red (NIR) spectroscopy is now recognized worldwide as technology appropriate for industrial quality and process control. NIR is used not only for quantitative analysis but also for identification of materials anywhere in the formulation process: raw materials, intermediates, and finished products.

Additives for lubricants like oxidation inhibitors, corrosion inhibitors, and metal deactivators are highly specialized chemicals. Their funciton is an important part of the lubricant behaviour. Some are readily distinguished because their NIR spectra are different. Those similar compounds with nearly identical NIR spectra require special chemometric treatment in order to identify them as the proper substance. These same chemometric methods can be used to qualify the material as the specified grade and quality. NIR instrumentation and software capable of this qualitative analysis were presented.

Along with all the usual benefits of NIR analysis, this identification and qualification method provides a more complete quality assurance program for lubricant production.

### Fast and accurate determination of aromatics in jet fuel using NIR technology

**Herman He**
*LT Industries, Inc., 6100 Executive Blvd., Suite 800 Rockville, Maryland 20852, USA*

The jet fuel blending process requires close monitoring of the aromatics concentration to assure the fuel quality and the optimum yield and throughput. The traditional aromatics measurement with the FIA (Fluorescence Index Analysis) method is destructive, time consuming, and has a poor measurement repeatability. A fast and accurate alternative is obviously required.

This paper presented a successful NIR application in the determination of aromatics of the jet fuel. Compared to the FIA method, the NIR technique is fast, precise, accurate, and nondestructive. Also, the NIR spectrometer is capable of operation in a configuration with multiple optical channels monitoring multiple process streams.

### A high throughput imaging fluorometer for real-time analysis of microtitre plate assays

**John F. Turner II**
*Department of Chemistry, University of Pittsburgh, Pittsburgh, PA 15260, USA*
**and Patrick J. Treado**
*Chemicon Inc., 7301 Penn Ave., Pittsburgh, PA 15208, USA*

Conventional microtitre plate readers analyse local chemistries in each well of the plate by sequentially rastering an optical reader head assembly from well to well or by sweeping a one dimensional detector array across the plate. The temporal resolution of this process is limited primarily by the sequential scanning process and number of wells. As a result, monitoring dynamic processes within all wells simultaneously is not feasible.

To address the limitations of conventional reader technologies the authors have developed a high throughput chemical imaging fluorometer that combines charged coupled device (CCD) detection with novel liquid crystal tunable filter (LCTF) imaging spectrometers to select the excitation and emission wavelengths under digital electronic control. A spectral image of the entire plate is captured by each detector reading making the acquisition time independent of the number of wells and the well geometry. Complete excitation, emission, and reflectance spectra can be acquired without the use of filter wheels or inefficient dispersive monochromators. The development of the microtitre plate imaging fluorometer and the role of chemical imaging in bioanalysis, bioprocess monitoring, and drug discovery were discussed.

### Automated analysis of extractable petroleum hydrocarbons (EPH) with solid phase extraction (SPE DISKS)

**Michael C. Janes**
*Horizon Technology, 8 Commerce Dr., Atkinson, NH 03811, USA*

The Extractable Petroleum Hydrocarbons and Petroleum Hydrocarbons (EPH/PHC) method is typically used in place of a Total Petroleum Hydrocarbon (TPH), generating more specific data. The samples are extracted with three consecutive shakes of methylene chloride (60 ml/shake). Solid Phase Extraction (SPE) as an alternative, offers many advantages over traditional Liquid Liquid Extraction (LLE).

SPE reduces the extracting solvent from 180 ml to 15 ml, while emulsions are eliminated, resulting in higher recoveries. The concentration step, glassware usage and overall extraction time is minimized. Automating SPE allows the laboratory to reduce labour and cost significantly while increasing the quality of data and reproducibility. The modular design allows multiple samples and methods to be run at the same time, increasing productivity.

This paper discussed analytical recovery data produced using the SPE-DEX 4700 (47 mm disk), manual 90 mm disk, and LLE for PHC and EPH samples. Issues of disk plugging and sample preparation were addressed.

### Automated solid phase extraction of drugs of abuse: a cost-effective approach

**David O. Hall and Dennis D. Blevins**
*ANSYS, Inc., 2 Goodyear, Irvine, CA 92618, USA*

Drug testing laboratories require precise, rapid and cost-effective procedures to perform analyses. Historically, laboratory technicians have performed all sample extraction steps of these procedures manually. SPEC solid phase extraction (SPE) columns offer significant time and reagent savings compared to both conventional liquid/liquid and packed-bed solid phase extraction, but sample preparation is still labour intensive.

Automated sample processing devices for SPE are a logical alternative, preparing samples unattended, freeing technical staff to perform other tasks. Until recently, these devices have been tailored for use in high-throughput laboratory situations. Laboratories performing a relatively small number of extractions (fewer than 100 samples per day) often cannot justify the use of expensive and complicated automated sample processing equipment. The Zymark RapidTrace offers low initial cost, simplicity of operation, and the capability of incremental expansion to keep pace with increasing workload.

Automated extraction protocols for the SAMHSA 5 (benzoyl-ecgonine, THC-COOH, amphetamine/methamphetamine, PCP and morphine/codeine) have been developed and were discussed. The presentation reported on work completed, discussing the approach taken during method development and the results achieved. Data presented included absolute recovery, linearity, carry-over, LOD/LOQ and sample throughput.

### The performance of parallel automated SPE for pharmaceutical and clinical bioanalysis

**Francois Qian, Christophe Fraudeau, Eric Verette, Genevieve Luck**
*Gilson Medical Electronics (France) S.A., 72 Rue Gambetta, BP 45, 95400 Villiers-le-Bel, France*
**and George Hutchinson**
*Anachem Ltd, 20 Charles Street, Luton LU2 0EB, Bedfordshire, UK*

Solid Phase Extraction is the technique of choice for the preparation of biological samples such as plasma, serum or urine before HPLC, GC/MS or LC/MS/MS. For this latter analytical technique, which typically results in extremely short run times, the throughput of the sample preparation step is an important issue.

The SPE processing time per sample can be optimized, but it is not possible to decrease it below a certain limit because flow characteristics through the sorbent material and extraction kinetics are limiting factors. Parallel (or multichannel) processing thus appears the best solution for dramatically increasing sample throughput.

The performance of parallel SPE workstations must be evaluated using several new criteria such as the actual sample throughput, between-channel relative standard deviation, sample carry-over, and the transfer of the extract to analytical instruments.

A new parallel Solid Phase Extraction workstation, the ASPEC™ XL4, was presented. With use of a multiprobe design, ASPEC XL4 processes four samples at a time, providing a sample throughput of 25 to 50 samples per hour. The performance of the instrument was reviewed using the above mentioned criteria and is illustrated with pharmaceutical and clinical applications.

### Automated on-line coupled SPE-capillary GC applications to ground water pollution control

**Adriano Trisciani, Fausto Munari, Pier Albino Colombo and Sorin Trestianu**
*CE Instruments, Strada Rivoltana, I-20090 Rodano, Milan, Italy*

Analysis of micropollutants in water requires a concentration step prior to gas chromatographic analysis. Presently this is mostly achieved by Solid Phase Extraction (SPE). On-line coupling of SPE to capillary GC (SPE-CGC) has some important advantages:

- Automatic analytical procedures.
- Use of the whole amount of available extract.
- Increases sample throughput by achieving simultaneously the extraction and analysis steps.

The paper described the analyses of organophosphorous pesticides (OPPs) in surface waters by the on-line coupled SPE-GC technique, using a fully automated computer controlled analyser. The instrument is a CE Instruments dedicated capillary GC system provided with a loop type interface and coupled on-line with an

SPE autosampler (Gilson-France). The equipment is piloted by a PC which also represents the data system.

The performances were illustrated by the analyses of OPPs in river water at concentrations lower than 0.1 μg/l (which is the maximum admitted value for drinking water in the European Community).

The water samples (up to 10 ml) are pumped through the SPE cartridge. After cartridge drying (15 min nitrogen flow 300 ml/min) pollutants are eluted with a mixture of MTBE/Dioxan 90/10 and transferred on-line to the gas-chromatograph using a loop type interface. Dioxan (co-solvent) was added to the MTBE eluting solvent in order to widen the application range of the loop transfer technique towards more volatile pesticides Mevinphos and Trichlorfon. Moreover it improves the peak shapes and the SPE separation.

Standard mixtures of pesticides were prepared with HPLC-grade water spiked with 8 OPPs at a concentration of 10 and 100 ppt. The standards were used to check repeatability, recovery, linearity and minimum detectable amount (MDA). The lowest limit of detection for the OPPs ranges within 10–20 pg. If 10 ml of sample is injected pesticides concentrations as low as 1–2 ppt can be detected.

### Screening for more than 400 pesticides by GC/AED

**Philip L. Wylie and Bruce D. Quimby**
*Hewlett-Packard Co., 2850 Centerville Rd., Wilmington, DE 19808-1610, USA*

A method has been developed that can be used to screen for more than 400 pesticides by combining a compound's absolute retention time under 'locked' chromatographic conditions with its elemental content. Chromatographic locking is a procedure used to adjust for changes in column length, diameter, or film thickness so that the retention times of the analytes match known values. Retention time locking works because modern capillary columns, combined with electronic pneumatic control of carrier gases, make retention times under specified GC conditions much more predictable. The elemental content of a chromatographic peak is determined by use of gas chromatography with atomic emission detection (GC/AED); the AED is selective for any element in the periodic table (except He) and can be used to determine which elements are present in a GC peak.

A database of more than 400 pesticides has been created that contains each pesticide's molecular formula and retention time on a 30 m × 0.25 × 0.25 μm HP-5MS column. The retention times were obtained from the literature and were translated from the published column to the HP-5MS column under both GC/AED and GC/MS conditions using HP's Method Translation Software.

To identify a pesticide, the user enters a peak's retention time and elemental content (elements known to be present in the compound and those known to be absent) into a simple form. The software then chooses those pesticides which fall into a small retention time window and have the correct elemental content. Confirmation is accomplished by calculating the peak's elemental ratios from the AED data or by use of GC/MS.

### Determination of selenium species in selenium enriched garlic using high performance liquid chromatography with inductively coupled plasma mass spectrometric detection

**Honghong Ge, Julian F. Tyson, Peter C. Uden, Xiao-Jia Cai**
*Department of Chemistry, University of Massachusetts, Amherst, MA 01003-4510, USA*
**Howard E. Ganther**
*Department of Nutritional Sciences, University of Wisconsin-Madison, WI 53706, USA*
**Eric Block**
*Department of Chemistry, SUNY-Albany, NY 12222, USA*
**and Eric R. Denoyer**
*Perkin-Elmer, 761 Main Ave., Norwalk, CT 06897, USA*

Garlic has been used as folk medicine for more than 500 years. The active component is the micro-nutrient selenium. Selenium deficiency causes skeletal and cardiac muscle dysfunction. Selenium-enriched garlic (fertilized with selenite and selenate) inhibits mammary cancer better than regular garlic. Determining the selenium species is important to elucidate the biochemistry of selenium in garlic and to find out the best form for treatment of diseases caused by selenium deficiency. Chromatography with non-specific detection is problematic due to the likely interference from high concentrations of sulphur analogues. This interference could be overcome by the use of element specific detection. Ion chromatography and reverse phase liquid chromatography have been coupled with ICP-MS detection. Selenite, selenate, methyl-selenocysteine, selenomethionine, and propyl-selenocysteine have been identified in garlic and other vegetables. Several unknown selenium species were observed.

### On-line silylation of short chain fatty acids followed by headspace GC/MS

**Yuwen Wang, Harold M. McNair**
*Department of Chemistry, Virginia Tech, Blacksburg, VA 24061, USA*
**and Maha Khaled**
*Cultor, Food Science, Technical Service Division, Eastern Point Rd., Bldg. 296 Groton, CT 06340, USA*

Short chain fatty acids are difficult to detect at trace levels in gas chromatography due to their strong polarity and reactivity. Derivatization of these acids before chromatography has been used for decades, however, the tedious manual steps of derivatization, dehydration and extraction detract greatly from the usefulness of this procedure.

A quick, simple and selective on-line silylation has been developed for the rapid determination of short chain fatty acids. The acids (C2 to C8) were on-line reacted with BSTFA (N,O-Bis(trimethylsilyl)trifluoroacetamide) in a sealed headspace vial and detected by GC-MS. The reaction conditions including reaction solvent, time and temperature are investigated. Various headspace injection techniques (split vs. splitless) were tried to enhance the detection sensitivity, peak resolution and efficiency. Hydrolysis products of a low calorie triglyceride comprising short chain and long chain fatty acid residues, and its hydrolysed derivatives were analysed by this new method. The technique gave a detection limit of 100 pb for the different acids considered and a precision of 5–10% RSD.

### Neuropeptide profiling of individual invertebrate neurons

**Jonathan V. Sweedler**
*Department of Chemistry and the Beckman Institute, Urbana, IL 61801, USA*

In many marine molluscs, such as *Aplysia californica*, multiple neuroactive peptides are found in individual identified neurons. In order to better understand chemical communication between neurons, the ability to follow the microenvironment in and around the neuron with spatial, temporal and chemical resolution is desired. Capillary electrophoresis (CE) is ideally suited for taking snapshots of the chemical composition at a specific local site but does not provide dynamic (time-based) information; microelectrodes provide dynamic but limited chemical information. While no single analytical technique provides all required information, we are developing methods that provide multi-dimensional information to profile the neuropeptides in single cells.

One approach modifies CE to add time resolution. The author used a sampling capillary placed near the cells of interest to sample the release as a function of time. The outlet end of this sample capillary deposits this material across the entrance to custom constructed rectangular electrophoresis system. In this way, a series of snapshots of the changing chemical composition occurring at the inlet end of the sampling capillary is obtained; in essence, one axis of the channel is a time axis and the other provides chemical information based on electrophoretic migration time, with the size and flow rate at the inlet capillary dictating the spatial sampling area. The utility of this method was demonstrated by assaying neuropeptide release from the bag cell neurons of *Aplysia*, a common neuronal model. These neuroendocrine cells contain a number of physiologically active peptides and can be electrically stimulated to release peptides over a many minute release event. Detection can be accomplished by radiolabelling the cells with $^{35}$S-methionine so that all the newly synthesized methionine containing peptides are radiolabelled. Another capillary-based detection method uses fluorescence after derivatizing the sampling capillary effluent before it enters the channel.

The capillary sampling scheme can be adapted to collect cellular release fractions for subsequent mass spectrometric analysis using matrix-assisted laser desorption ionization (MALDI) time-of-flight mass spectrometry. A separation is not even needed with MALDI, as individual cells can be isolated and placed directly on the MALDI target; with appropriate rinsing to remove the marine salts, high quality mass spectra are obtained. Alternatively, cells can be cultured on glass slides, and by aiming the laser to specific areas of the cell, mass spectra are obtained from spatially resolved cellular sections. The information obtained from these techniques was compared for spatial, temporal and chemical profiling of a series of peptidergic invertebrate neurons.

### New horizons in flow injection atomic spectrometry

**Julian F. Tyson**
*Department of Chemistry, University of Massachusetts, Box 34510, Amherst, MA 01003-4510, USA*

Since the first papers 18 years ago, there have been steady developments in the use of flow injection (FI) techniques with atomic spectrometry (AS) such that the use of some variant of FI for the automated pretreatment and/or presentation to an atomic spectrometer is now a commercially available option for use in routine laboratory situations. While there has been some debate over the definition of 'flow injection', there is a very practical basis for an interest in the use of whatever FI is with AS techniques, namely that FIAS methodology is a way of saving money for any laboratory. In the atomic spectrometry laboratory, FI is probably best regarded as a technology for the automation of sample pretreatment with direct coupling to the instrument.

What are the likely future developments that are on or just over the horizon? Perusal of the regular review literature suggests that large numbers of enthusiastic researchers contribute to this topic of FIAS, and it is relatively easy to project what the immediate future holds in terms of research output. Most research papers will be concerned with AAS. The majority will be concerned with applications involving FAAS and most of these will be applications of some form of preconcentration and matrix removal, most likely by solid phase extraction. An almost equal number of papers will be concerned with chemical vapour generation followed by atomization in quartz tubes, with a small number of applications employing trapping of the volatile derivative inside a graphite furnace atomizer. A substantial number of papers will involve the determination of mercury.

Activity in relation to plasma spectrometry will be relatively limited, mainly because commercial instruments cannot handle transient signals, either in a single element or multi-element mode. This is the single most pressing need in instrument development. There is relatively little interest, at present, in other transient signal sample introduction devices such as laser/spark ablation, or electrothermal vaporization, so it will be the need for speciation measurements that will drive developments in transient signal data processing. In addition to the various ways in which chemistry can be applied by FI techniques to yield species dependent information, various chromatographic and electrophoresis techniques will be increasingly used. It is likely that solid-phase reagent chemistry will feature as the basis for speciation by FIAS.

Many trace analyses are currently limited by the purity of the reagents needed. Flow injection techniques will provide the means to break through this barrier. Some interesting developments in hydride generation were presented.

### Sequential injection and flow injection with drops and films

**Purnendu K. Dasgupta and Hanghui Liu**
*Department of Chemistry and Biochemistry, Texas Tech University, Lubbock, TX 79409-1061, USA*

A liquid drop has some unique features, namely, reproducibility, renewability, and the lack of containment walls. The value of these features is highlighted through a series of novel liquid drop based systems: truly renewable gas sampling interfaces, windowless optical cells and reaction vessels for flow injection turbidimetry, and solvent extraction systems with nested drop arrangements. The use of a drop minimizes sample/reagent consumption and like the dropping mercury electrode of Heyrovsky, allows a fresh reaction surface for every sample. Drops are of particular value in solving problems in biphasic systems.

### Flow injection based renewable sensor systems (FI-RS)

**Jaromir Ruzicka and Gary D. Christian**
*Department of Chemistry, Box 351700, University of Washington, Seattle, WA 98195, USA*

Fouling of sensing surfaces has been the Achilles' heel of chemical sensors that prevents their widespread practical use. In addition, the present concept of chemical sensors severely limits the choice of sensing mechanisms, since the suitable chemical reactions must be fully reversible (as otherwise the sensor can be only used once) and the sensing surfaces must be stable throughout the sensor lifetime (to avoid need for frequent recalibration). That is why the paradigm has to change by recognizing that chemical sensor alone will never fulfil the requirement on ideal functionality. Indeed, in the future we shall be using *sensor systems* based on sequential injection (SI) methodology, whereby calibration, sample conditioning and sensing surface renewal will be carried automatically whenever needed.

The lecture opened by comparing merits of FI and SI, and progressed to a concept of using microbeads as renewable sensing surfaces. The principle of bead capturing and monitoring was illustrated by several configurations of the jet ring (JR) cell, as dictated by requirements of electrochemical, reflectance, absorbance or fluorescence measurements.

The active volume of JR cell is about 5 microlitres, that accommodates between 10 to 20,000 beads (beads in the range of 40 to 150 microns). A wide variety of bead materials has been used including Dowex-1, Dowex 50, Sephadex 6B, porous glass, Cytodex, Oxirane and glassy carbon. The surface functional groups ranged from ion exchangers to covalently bound enzymes, Protein A and antibodies. The analytes comprised herbicides, glucose, ethanol, trace metals as well as strong acids dissolved in organic solvents.

At this early stage the versatility of FI-RS is its most attractive feature as it offers access to a vast arsenal of (irreversible) reagent chemistries and carrier beads developed by generations of chemists and biochemists. In a long run, when perfected, FI-RS will allow to eliminate the flaw in the present concept of the chemical sensor technique; the apparent need for a sensing layer permanently attached to the surface of a transducer.

### New horizons in flow injection–capillary electrophoresis

**Bo Karlberg and Petr Kuban**
*Department of Analytical Chemistry, Stockholm University, S106 91 Stockholm, Sweden*

A new interface for coupling flow injection analysis (FIA) and capillary electrophoresis (CE) has been developed. A sample plug injected in the FIA part of the system is carried by an electrolyte stream towards the interface. The first end of a capillary is positioned in a flow-through channel inside the interface together with a platinum electrode. The other capillary end and a second platinum electrode are immersed in a liquid reservoir situated outside the interface. A constant high voltage is applied across the two platinum electrodes. An FIA system is connected to the flow-through channel of the interface. When a sample plug, injected into the FIA system, passes by the first capillary end, a minor fraction of the sample is introduced into the capillary and an on-line separation is performed. A UV detector is placed close to the second capillary end. The FIA-CE interface enables multiple injections so that a high sampling throughput can be acieved (up to 150 samples per hour). The interface was tested by running a standard mixture of nine common anions. The repeatability was about 2% (r.s.d.).

On-line dialysis and gas diffusion performed in the FIA system can be integrated with the CE system via the specially designed interface. Samples are continuously pumped into a dialysis or gas diffusion unit and the outgoing acceptor stream containing the analytes is allowed to fill the rotary injector in the FIA part of the system. Multiple sample injections are enabled in one electrophoretic run and the entire analytical procedure can easily be mechanized. The repeatability is in the range 1·6–3·3% ($n=7$). A wide range of real samples with complicated matrices (milk, juices, wine, vinegar, liquors from the pulp and paper industry) was successfully analysed without any off-line pretreatment using the fully mechanized system. So far only small anions have been determined with this technique. Studies are in progress to integrate further pre-treatment procedures and to include determinations of cationic species.

### Miniaturized flow injection analysis with an integrated electrochemically-driven micropump

**Jing Ni, Chuan-Jian Zhong, Shelley J. Coldiron and Marc D. Porter**
*Microanalytical Instrumentation Center, Ames Laboratory-USDOE, and Department of Chemistry, Iowa State University, Ames, Iowa 50011, USA*

One of the key steps towards the miniaturization of fluid flow-based analytical methodologies, such as flow injection analysis (FIA) and liquid chromatography, is the integration of micropumping and injecting systems. Most of the recent approaches to this end rely on micropumps and microvalves that require high voltage power supplies. The results from the preliminary development of a miniaturized FIA system that is based on a novel, low power, electrochemically-driven micropump were presented. The basis of pump actuation relies on electrochemically-induced surface tension changes at the electrolyte/mercury interface, resulting in a 'piston-like' pumping process devoid of mechanically moving parts. The flow rates and pumping displacement volumes have been studied as a function of the amplitude and the frequency of the applied voltage. Flow rates in the range of 5 to 1000 μl/min, displacement volumes as high as 10 μl per stroke, and stroking frequencies up to 50 Hz have been demonstrated using a capillary column design for the FIA system with both bore-type and 'valveless' nozzle/diffuser checkvalves employed for directional flow. Results using electrochemical, optical, and video detection formats were presented as concept demonstrations of the integrated FIA system for acid–base titrations and metal–ligand complexations. These results were compared with those obtained using conventional pumping approaches for performance evaluations.

### ***In situ*** **fibre spectroscopy of hydrocarbon contaminants in natural water and soils**

**Christopher M. Stellman, K. J. Ewing and F. Bucholtz**
*Fiber Optic Environmental Sensors Section, Code 5603.3, Naval Research Laboratory, Washington, D.C. 20375-5338, USA*

Hydrocarbons are ubiquitous contaminants in natural water and soils. Sources range from anthropogenic pesticides, discarded solvents and petroleum discharge to natural oils and humic acids. Traditionally, hydrocarbon analysis is performed by laboratory or field gas chromatography-mass spectrometry or liquid chromatography-ultraviolet fluorescence. These chromatographic methods are hampered by 20+ minute sample analysis times (not including any required sample preparation), sample handling and transport problems for laboratory analysis, and instrument stability problems for field systems.

In contrast, fibre-optic spectroscopy has been used to remotely monitor numerous chemical and physical phenomena. Small fibre-optic probes are resistant to severe environments (e.g. high temperatures, mechanical shock, etc.) and provide an ideal interface between the analyte of interest and spectroscopic hardware. Also, fibre-optics are flexible and can transmit light over long distances (100 m) with little attenuation. This can isolate sensitive electronics (sensors) from the point of detection and allow for remote monitoring of harsh and/or spatially confined locations. Finally, because fibre-optic probes are inexpensive and easily multiplexed they provide a means of simultaneously monitoring numerous sample sites.

The presentation described preliminary work in the development of a fibre-optic Raman spectroscopy system for *in situ* monitoring of hydrocarbon contaminants in natural waters and soils. The instrument's design was described in detail and preliminary results were presented. In addition, the performance of the instrument was compared and contrasted with more conventional analytical methods. And finally, the potential of the instrument to contribute to the remediation and characterization of hazardous sites was discussed.

### **Quick screening for drugs of abuse by fast gas chromatography**

**Erin Wiley and Anthony Andrews**
*Center for Intelligent Chemical Instrumentation, Clippinger Laboratories, Ohio University, Athens, OH 45701-2979, USA*

Applications for fast gas chromatography (GC) are becoming more common as analysts take advantage of the benefits offered by this technique. One area in which fast GC may be particularly useful is for the qualitative screening of a large number of samples for a simple positive/negative result. Such screening occurs routinely for drugs of abuse such as heroin, cocaine and marijuana in forensic laboratories.

Using a conventional GC, modified with a carbon dioxide based cryotrap/thermodesorption inlet system a number of compounds of forensic interest have been studied. Commercially available mixtures of opiates, barbiturates, depressants and stimulants have been separated in under two minutes.

The major drugs of abuse are cocaine, heroin and marijuana. Chromatograms will be shown detailing the separation of these compounds from their metabolic products in under two minutes. The potential for one short chromatographic run which could tentatively screen for these three compounds and their breakdown products will be discussed.

Details of the experimental procedure were given and results were presented for all mixtures studied. In addition preliminary data on the sampe preparation protocol suitable for coupling with the fast GC analysis were given. The possibility of direct injection for aqueous samples such as urine was discussed. A complete positive/negative screening system for the major drugs of abuse with results available in under 5 minutes being the ultimate goal.

### **Selected applications of programmed temperature injection systems in capillary GC**

**Hans-Gerd Janssen, Mark van Lieshout and Carel Cramers**
*Eindhoven University of Technology, Laboratory for Instrumental Analysis, P.O. Box 513, 5600 MB Eindhoven, The Netherlands*

Sample introduction is very often the most difficult step in a gas chromatographic analysis. In particular this is true for samples that require sample pretreatment prior to introduction in the gas chromatographic system. In the past a wide variety of techniques for sample pretreatment has been developed. Basically these techniques can be classified as thermal treatment procedures such as thermal desorption, pyrolysis or head space techniques or as liquid treatment procedures as solid phase (micro) extraction.

In the present contribution the use of the programmed temperature injector (PTV) as a universal interface for on-line thermal- and liquid-sample pretreatment procedures was discussed. Large volume injection is the key to on-line combination of liquid sample pretreatment and capillary GC analysis. In the lecture the principles of PTV large volume injection were briefly discussed and the use of the PTV injector as an interface in on-line analysis and clean-up of liquid samples were described. A fully automated system for on-line solid phase extraction with capillary GC analysis of environmental and forensic samples was shown.

### Developing robust HPLC separations using an automated method development system

**Michael W. Dong, Yury Rozenman, Gene Dalton and Frank Vandemark**
*The Perkin-Elmer Corp., Chromatography Division, 761 Main Avenue, Norwalk, CT 06859-0250, USA*

This paper detailed the use of a rapid method development and mobile phase optimization system for HPLC. Simulation data were used initially to predict starting conditions. The methods development software allows automated searches of quaternary solvent space. Results are mapped graphically to reveal the best mobile phase composition. Additionally, automatic peak tracking and chromatographic interpolation routines are included. The development of a robust stability indicating assay for an antiinflammatory oxaprozin drug substance and drug product was used as an example.

### Automated solvent blending enhances HPLC performance

**Donna Dion-Wall, John Morawski, Jeanne Li and Art Sims**
*Waters Corporation, 34 Maple Street, Milford, MA 01757, USA*

In the past, a dial-a-mix, or an instrument controlled approach to solvent proportioning has caused problems with reproducible chromatographic results. In HPLC, retention times are used for peak identification where the standard and unknown must elute at the same time. Some of the possible causes of irreproducible results associated with dial-a-mix are non-reproducible solvent proportioning, outgassing and insufficient mixing. The Waters 2690 Separations Module represents a new dimension in reproducible HPLC performance. Incorporating a design which consolidates and controls all the critical fluidic functions of the LC process, the Waters 2690 provides reproducible performance from unit to unit, lab to lab, and chromatographer to chromatographer. Accurate solvent proportioning eliminates the variability of premixed solvents and solvent handling and allows for easier, more reproducible method and system transfers. The advanced Synchronized Composition Control, SCC, software coordinates the gradient proportioning valve with the piston position and flow rate for reproducible gradient performance. This overall design results in accurate and reproducible automated solvent blending and gradient formation.

The ability to accurately program and reproducibly deliver any desired solvent composition with the Waters 2690 Separations Module has been demonstrated with several experiments comparing the Waters 2690 to conventional systems. One experiment uses alkylphenones to demonstrate the retention time shifts with changes in the solvent proportioning of methanol and water over 20 different compositions in 1% changes. Another experiment separates soft drink additives to demonstrate the chromatographic sensitivity of thes ionic compounds with slight solvent changes. These experiments, along with other data illustrate that reliable results can be a reality with this automated solvent blending technique.

### Use of database applications in improving operational efficiency

**Melissa V. Demaray and Andrew Grygiel**
*Hewlett Packard Company, Palo Alto, CA 94304, USA*

Efficient management of the modern analytical laboratory necessitates timely and complete availabilty of data and information to laboratory processes, and to laboratory decision makers. An information infrastructure must be designed to support both the analytical work and the management decisions related to operational efficiency, quality and cost control. Database technologies play a vital role in improving the ease and speed of access, management and use of laboratory data and information.

Database management systems are tools used for collecting and organizing data so that it is easily retrieved by users and applications. It is database applications, however, that provide the meaningful access to data and information that is stored in the database, thereby enabling quick, informed decision making. When both the database management system and the applications which support specific tasks are designed to support multiple users, special consideration must be given to administration, security, and user privileges.

Examples of database applications that improve efficiency of laboratory operations and people include: the tracking of instrument performance and consumables for the purposes of predictive maintenance and replacement; tracking of instrument utilization and types and numbers of analyses for predictive planning purposes; and trending of analytical results to support changes in production or process improvement.

### Cost management as the basis for modern laboratory operation

**David Berwanger**
*Sabercat Software Ltd., PO Box 760, Folsom CA 95763-0760, USA*

All laboratories face pressure for increased productivity while continuing to improve financial performance. Decreased requirements for environmental services, restrictive budgets or public sector competition are but several factors that contribute to this burden. Even R&D facilities, traditionally insulated from the need to perform analyses at the most competitive cost are being scrutinized for alternative analytical strategies that better address the issue of economy.

The traditional method to solving this problem was a mechanical approach: purchase of increasingly sophisticated, faster, more sensitive and often expensive analytical instrumentation and/or automation technology that sometimes lowers the analytical cost. While this approach may temporarily solve the productivity problem, it also requires a draw on scarce financial resources to implement. A lower cost alternative would place greater emphasis on modern *business management* techniques, beginning with cost analysis as the basis.

The development of business software for laboratory management has not progressed as rapidly as microprocessor driven instruments used in the laboratory. Surprisingly, costing software has been available for decades in traditional manufacturing environments, but rarely modified for adoption into the laboratory operation. Greater capacity, faster throughputs and increased analytical sensitivity will not solve the problems of shrinking budgets and diminished financial performance in a mature market. Without properly identifying the cost factors associated with testing, many laboratories will continue to struggle financially.

A method and application software for deriving and appying test-cost information in *any* testing operation was described, using cost analysis as part of a management solution to enhance laboratory financial performance. The application software utilizes a standard PC operating system and database widely available and in use in the laboratory environment. Standard reports identify the value of the analysis and each of the components, the per-sample value of the instrumentation, most financially efficient batch size, etc.

### Lab management/data analysis

**Steve Bernet**
*Labtronics Inc., 95 Crimea Street, Guelph, ON, Canada N1H 2Y5*

Many automated laboratories are using new software tools to create intelligent instrument interface workstations. These workstations perform an array of functions beyond the basic function of transferring a result to a LIMS database. Some of the functions of these new data handling systems are discussed including their capability to:

- Acquire work lists from LIMS.
- Generate a complete auto sampler sequence file.
- Upload auto sampler sequence files to instrument systems.
- Acquire instrument data from manual instrument systems.
- Acquire data from automated instrument systems.
- Combine data from multiple instrument sources.
- Perform calculations.
- Perform data manipulations, including string substitutions and date conversions.
- Query LIMS databases to retrieve information, such as testing limits.
- Flag out-of-range conditions and exceptions.
- Invoke external algorithms to analyse data and verify results against quality control protocol rules.
- Permit test results to be reviewed and approved before committing to LIMS.
- Archive all original instrument data and processed data.
- Maintain system security and create audit logs.

### Managing productivity and quality in an analytical laboratory in a developing country

**W. H. J. de Beer**
*Department of Chemistry and Physics, Technikon Pretoria, PO Box 56208, Arcadia 0007*

In the developing countries with high population growths, high inflation rates, low gross national products per capita, the creation of wealth is of the utmost importance. Science and technology are accepted to be the real creators of wealth, and the analytical laboratory must also be regarded as a potential wealth creator. Wealth can be best created if you can compete in the global village. To improve your competitiveness, you must improve your productivity.

Productivity implies to be efficient and effective. The analytical laboratory is efficient if it can convert its resources into services, and it is effective if the services can satisfy the needs of customers, both internal and external.

From an efficiency point of view, resource management is of the utmost importance and in this paper the management of laboratory personnel, instrumentation, materials, methodology, finance and markets were discussed.

From an effectivity point of view needs satisfaction implies the needs of the laboratory and of the customers. Needs can only be satisfied if wealth is created, both by the analytical laboratory and by the customer. Creation of wealth can imply quality, lowering cost, making a profit. In this paper the important relation between quality, productivity and wealth creation was emphasized.

### Flow injection analysis of fructose

**Fereshteh Haddadian, Christine A. Heenan and Neil D. Danielson**
*Miami University, Department of Chemistry, Oxford, OH 45056, USA*

Previously the authors have shown that zirconyl chloride ($ZrOCl_2$), is readily hydrolysed in water to form $ZrO(OH)^+$. $ZrO(OH)^+$ can react with sugars such as fructose, glucose, and sucrose to form fluorescence chelates which have excitation and emission wavelengths of 348 and 418 nm, respectively. Because the reactivity of glucose and sucrose is less even at an elevated temperature, fructose can be selectively determined in the presence of the other two sugars.

This reaction has been used for flow injection analysis (FIA) using a 15 cm × 4.0 mm polystyrene–divinylbenzene packed HPLC column as a mixing device. The fructose sample was injected at a flow rate of 0.1 ml/min which was maintained for 5 min. The flow rate was then increased to 1 ml/min for 2 min to elute the chelate peak quickly. The optimum column temperature for the reaction was found to be 100°C. Using 0.01 M $HClO_4$

with a 1% zirconyl chloride carrier, a linear calibration curve was obtained for 2 to 30 mg/l with a correlation coefficient of 0.994. A detection limit of less than 2 ppm was achieved.

To determine the specificity of the FIA method, an enzymatic determination of fructose was studied. D-fructose dehydrogenase (FDH) was used as a catalyst during the oxidation of D-fructose in the presence of ferricyanide:

$$\text{D-fructose} + 2K_3Fe(CN)_6 \xrightarrow{FHD} \text{5-keto-D-fructose} + 2K_4Fe(CN)_6$$

$$2K_4Fe(CN)_6 + \text{Ferric sulphate} \longrightarrow 2 \text{ Prussian Blue} + 3K_2SO_4$$

After a reaction time of 45 min, the absorbance of the Prussian blue at 660 nm was measured. A comparison between the FIA and enzymatic results for a variety of samples did confirm that the FIA method was highly specific to fructose. For example, the soft drink Sprite (after 1 : 500 dilution) was assayed by 3 different methods and compared to the label value of 62 g/l fructose. Standard spectrophotometry of the zirconyl–fructose chelate indicated 61 g/l while both the FIA and the enzymatic methods gave results of 63 g/l. The FIA method had the advantage of facile sample throughput over the other two procedures.

### Comparison of automated Soxhlet and microwave extraction methods for fats

**Kevin P. Kelly and Nancy L. Schwartz**
*OI Analytical Sample Preparation Products Group, 555 Vandiver Drive, Columbia, MO 65202, USA*

Application of heat to extraction of solids with organic solvents immensely speeds up the attainments of mass transfer. Soxhlet extraction is faster, for example, when the sample is contacted by boiling extraction solvent instead of condensate. Microwave technology (MAP, or microwave assisted process) makes extraction proceed even more rapidly by transferring energy directly to sample and reagents with radiation rather than heating through vessel walls. Additionally, rapid localized heating can cause temperatures above normal boiling points of reagents or solvents, thus contributing to faster reaction kinetics.

A polar substance (usually the solvent) is required to absorb microwave energy. Recent studies showed that a fat-laden matrix can absorb enough energy to 'render' triglycerides into a non-polar solvent during open-vessel MAE. Non-polar solvents are needed to obtain results equivalent to traditional fat extraction methods, and they produce cleaner extracts of food matrices. Open-vessel MAE is investigated for determination of fat content in matrices such as meats, nuts, feed and grain.

Open-vessel microwave assisted extraction (MAE) is explored as an alternative to closed-vessel MAE or automated Soxhlet extraction. Extraction vessels at atmospheric pressure were placed in a focused microwave beam. Extraction is very rapid and requires only enough solvent to adequately contact the sample. Furthermore, good yields do not always depend on efficient absorption of microwaves by the solvent. For example, crushed rock core (containing about 3% petroleum) from a drilling operation was processed using open vessel MAE (10 grams of sample in 15 ml of solvent). Analysis of extraction aliquots (GC/FID) showed no increase in yield after 10 minutes of microwave exposure whether the solvent was methylene chloride or *n*-hexane. Extraction in hexane was rapid, even though hexane barely became hot enough to boil.

### Quantitative image analysis methods for automated near-infra-red fluorescence immunoassay

**John W. Silzel, Tsong Tsay and Robert J. Obremski**
*Beckman Instruments, Inc., 200 South Kraemer Boulevard, Brea, CA 92822-8000, USA*

Charge coupled device (CCD) detectors provide high sensitivity, linearity, dynamic range, and spatial resolution. These devices have made possible the collection of image data at low light levels, not only in astronomical and surveillance applications, but in fluorescence microscopy. The authors' laboratory conducts fluorescence immunoassays on 100–200 micron diameter zones, ink-jet printed onto a two dimensional film. Using this approach, it is possible to image hundreds of assay endpoints in a single CCD image. To reduce scattered light backgrounds, and concentrate laser energy in the analytical volume, a laser diode is prism coupled to the film, which forms a planar waveguide. Fluorescence from near-infra-red dyes on the surface of the film is then excited by means of the evanescent wave. At the assay endpoint, emissions from each zone are proportional to the quantity of analyte bound in that zone.

Image data are high in information content, but this complexity and the large memory storage requirements create a processing challenge given the high throughput required of clinical assay systems. Methods are needed for the rapid automated reduction of image data to quantitative analytical results based on the intensity of the imaged zones. Unfortunately, many of the present generation of image processing tools have been developed to perform qualitative enhancement of the image features for visual aesthetics, counting, or classification rather than multiple quantitative analyses. Tools and methods are needed which exploit the excellent photometric performance and large linear dynamic range available from the scientific grade CCD.

Simple algorithms for the automated location, selection, quantitation, and analysis of quantitative image data will be presented, with particular emphasis on automated immunoassay by photometric intensity measurements. Robustness of the methods in the presence of noise, image artifacts and 'clutter' was demonstrated using actual assay data. Quantitative precision and outlier detection were discussed.

The presentation concluded with a survey of slightly more sophisticated image filtration methods. This discussion emphasized the development of general filters for the identification of image features, the extraction of quantitatively significant features from the data, and suppression or elimination of image clutter and artifacts. Many of these filters can be developed using techniques analogous to those applied by chemometrics to the multivariate analysis of conventional spectroscopic data.

### On-line antibody mimetic receptor selection and analysis of small molecule combinatorial libraries by LC/MS

**Jerry Zweigenbaum, Ray Wieboldt and Jack Henion**
*Analytical Toxicology, Cornell University, 927 Warren Drive, Ithaca, NY 14850, USA*

Combinatorial chemical libraries offer great potential for identifying target compounds to develop new pharmaceuticals and industrial products. Ample importance has been placed on the synthesis of diversity in the creation of combinatorial libraries. However, libraries whether small or large are of little use unless appropriate selection and identification of compounds of interest are devised. Selection of large libraries has been made by screening techniques such as immunoassay. Identification has incorporated the use of coding systems into the synthesis of the libraries. Although elegant, these systems may not always provide the specificity desired. Alternative and supportive approaches to these procedures need to meet the requirement of handling numerous compounds in large libraries both as pooled and split samples. The use of affinity chromatography with LC/MS and LC/MS/MS may offer an alternative with high specificity both in the selection and identification process. Additionally, the ability to automate these techniques into a uniform procedure may fulfil the above requirement. To this end we have examined the use of the automated injection, immunoaffinity column selection, column switching to LC, and detection by mass spectrometry to select and identify potential targets in small benzodiazapine libraries. The major issues associated with such a system include non-specific binding, loading of valuable antibody or receptor, and sensitivity requirements of larger libraries. We have implemented an automated IAC/LC/MS/MS system to selectively capture and identify targets in 20–40 compound benzodiazapine libraries. Conditions required to obtain selective binding and strategies to address the above were discussed.

### On-line coupling of chromatographic separation methods with NMR spectroscopy

**Klaus Albert**

*Universität Tübingen, Institut für Organische Chemie, Auf der Morgenstelle 18, D-72076 Tübingen, Germany*

The structural assignment of unknown compounds separated by a chromatographic separation technique is often a problem. For routine analysis, peak recognition is mostly performed by a detector using the ultraviolet (UV) absorbance of the separated compound. Despite all advances in UV detector technology, the need for a universal detector still remains, especially for compounds with no chromophoric system. Nuclear Magnetic Resonance (NMR) spectroscopy with its superior stereochemical information content is one of the most powerful techniques for structural elucidation.

Therefore the hyphenation of chromatographic separation methods, such as High Performance Liquid Chromatography (HPLC), Gel Permeation Chromatography (GPC), Supercritical Fluid Chromatography (SFC), Capillary Electrophoresis (CE) and Capillary HPLC together with NMR spectroscopy is getting an increasing number of applications in pharmaceutical, bio-medical and polymer research. Whereas the current sensitivity values obtained in the hyphenated mode with modern NMR spectrometers are in the nanogram range, the resolution of the recorded NMR spectra approaches the quality of routine NMR spectra.

In most of the routinely performed hyphenated experiments NMR flow-cell detection volumes between 60–180 μl are used. These large volumes lead to a degradation of the chromatographic resolution. In on-line coupling experiments performed in the continuous-flow mode the introduction of a second dimension, the proton chemical shift, results in a partial compensation of the chromatographic peak dispersion. Here, coeluting peaks can be differentiated by their different chemical shifts. Chromatographic peak dispersion can be minimized by the use of capillaries for the chromatographic separation. Thus high-resolution 1D and 2D NMR spectra can be obtained by performing on-capillary NMR detection in the nanolitre scale. Despite the small detection volumes between 5–900 nl, the nanogram sensitivity range for 1D acquisition is not a problem.

In summary, the present high application power of the on-line use of NMR spectroscopy in separation chemistry was demonstrated by practical examples derived from the fields of pharmaceutical and polymer research.

### Intelligent sensor systems using semiconducting polymers and tin oxide devices

**Paul Wells, Paul Travers**
*AromaScan plc, Electra House, Electra Way, Crewe CW1 1WZ, UK*
**and Garry Merry**
*AromaScan Inc., 14 Clinton Drive, Hollis, NH 03049, USA*

Research in recent years has brought many advances in organic chemistry, electronics and computing which have made the electronic measurement of aromas possible. The present status of the various technologies was reviewed with particular emphasis on semiconducting polymers and tin oxide devices. A review of some of the information processing systems used for array sensors was given including classical statistical techniques and neural networks.

Developments by the University of Manchester Institute of Science and Technology (UMIST) and AromaScan have produced a commercial system based on semiconducting polymers that enjoys considerable international acclaim. The current AromaScan system was outlined. The performance of the device with regards to discrimination, sample throughput, repeatability and reproducibility was described. The response of the system to various classes of organic compounds (alcohols, aromatics, esters and heterocyclics) was reported. Application domains for the technology include fine chemicals, aerospace, cosmetics, food, beverages, biomedical, air monitoring, process monitoring, packaging and the petrochemical industry. These domains were illustrated by numerous examples.

### Determination of mercury in urine at ultra trace level by vapour generation atomic fluorescence spectrometry

**Reshan A. Fernando, Phyllis Elkins, Margaret M. Goldberg, Robert W. Handy**
*Research Triangle Institute, 3040, Cornwallis Road, RTP, NC 27709*
**and Bradley J. Collins**
*National Institute of Environmental Health Sciences, P.O. Box 12233, Mail Drop A2-02, RTP, NC 27709*

In support of a toxicological study to evaluate the effects of mercury (Hg) exposure on estrus cycling and hormone level in female Sprague Dawley rats, digestion and analytical methods were developed for quantitating total mercury in rat urine samples at parts-per-trillion level.

The digestion procedure involved an overnight treatment of homogenized urine at room temperature with 2.5 ml of concentrated nitric acid (trace metal grade) and 2.0 ml of $KBr/KBrO_3$ solution followed by the addition of hydroxylamine hydrochloride and diluting to 25 ml with deionized water. Sample analysis procedure involved reduction of inorganic mercury ions with tin(II)chloride to form gaseous elemental mercury, separation of gaseous mercury and detection by atomic fluorescence spectrometry.

Samples were recovered from control animals and animals exposed to mercury vapour at the 1, 2 and 4 $mg/m^3$ level. Animals were exposed to mercury vapour (1, 2, or 4 $mg/m^3$) or air (control) for 2 h/day for 11 consecutive days. Analytical results have indicated a good correlation between the total mercury measured and the number of days of exposure. Method validation results and sample analysis results were presented.

[This research was supported by NTP, Contract No. N01-ES-55386. This has not been subjected to Agency review and therefore does not necessarily reflect the views of the Agency, and no official endorsement should be inferred.]

### *In situ* monitoring of gaseous decomposition products of surveillance oven samples by FTIR

**Vane G. Smith, Jr and John M. Corbett**
*Indian Head Division, Naval Surface Warfare Center, 101 Strauss Avenue, Bldg 1864, Indian Head, MD 20640-5035, USA*

In an effort to automate and improve the Surveillance Oven Test (SOT) program, a novel method utilizing Fourier Transform Infra-red Spectroscopy (FTIR) is being developed. The SOT program is used to monitor the stability of nitrocellulose based gun propellants. The test consists of placing approximately 45 grams of the propellant in a ground glass stoppered bottle which is then placed in a 65.5°C oven until the sample begins to visibly generate reddish brown nitrogen dioxide ($NO_2$) fumes. The concentration of the $NO_2$ is never measured. No information at all is obtained on other gases of potential interest which are known to be present such as NO, $N_2O$, CO and $CO_2$.

The mid IR range (2500 to 1500 $cm^{-1}$) is ideal for observing and quantitating the concentrations of these gases. Specially modified bottles with ZnSe windows have been produced so that the gas concentrations can be monitored *in situ*. The spectrometer itself is located outside of the ovens in a temperature and humidity controlled chamber. The samples will be monitored in real time by interfacing the spectrometer to the sample bottles via mid-range fibre optic cables. The data obtained from various fuming samples were presented along with techniques for preparing standard gas mixtures and calibration curves.

### Differentiation of mercury and methylmercury by direct derivatization and supercritical fluid extraction with on-line spectral detection

**Yuh-Chang Sun, Jerzy Mierzwa, Yu-Teh Chung, Mo-Hsiung Yang**
*Department of Nuclear Science, National Tsing-Hua University, 30043 Hsinchu, Taiwan*
**and Chung-Sun Chung**
*Department of Chemistry, National Tsing-Hua University, 30043 Hsinchu, Taiwan*

From various reports on supercritical fluid extraction (SFE) of mercury species from solid materials it can be concluded that MeHg can be extracted out of a sample matrix relatively easy. Typically, a specific ligand is necessary for inorganic mercury extraction. A novel method of direct derivatization was developed by the authors of this paper.

A derivatization of inorganic mercury using sodium tetraphenylborate (STPB) was explored. After the reaction with STPB, a stable mercury signal was detected by atomic absorption spectrometry (AAS) system with heated quartz furnace coupled on-line with the SFE extractor furnace, confirming that increasing of the volatility of mercury ion (Hg II) by *in situ* benzylation can be performed. Owing to the fact that SFE extraction efficiency is highly sample dependent, the extraction efficiency of mercury from the samples of different matrices (silica gel, alumina and activated charcoal) was studied and discussed. Results of the optimization of main experimental conditions, including extraction pressure, time, temperature, STPB amount, etc. were presented.

### Non-invasive sampling and capillary electrophoresis for the determination of glucose in body fluids

**Valerie Zuccari, Rong Chen and Luis A. Colón**
*State University of New York at Buffalo, Department of Chemistry, Buffalo, NY 14260-3000, USA*

The analysis of biological fluids by non-invasive means is of great importance in clinical diagnosis. The use of noninvasive procedures offers the advantages of painless sampling, avoidance of problems associated with difficult intravenous access, and reducing the risk of infection. These advantages will encourage adherence to therapeutic treatments. For example, diabetics can keep account of blood glucose levels without skin rupture by needle penetration.

The authors are developing non-invasive procedures to sample biological fluids for clinical analysis. In particular, they are collecting tear and subcutaneous fluids which contain chemical information suitable for clinical diagnosis. The interest is the determination of glucose in these biological fluids. The samples are collected in micro- and sub-microlitre volumes. The analysis of the collected samples requires the use of capillary separation techniques because the sample quantities are small. Capillary electrophoresis (CE) with laser induced fluorescence (LIF) has been used to develop methodology for the analysis of the samples collected non-invasively. The samples are submitted to an enzymatic reaction scheme, producing a fluorescent compound that is proportional to the concentration of glucose in the sample. This fluorescent compound is then monitored using CE-LIF. The method is relatively fast, sensitive, and can accommodate small sample quantities. Detection of sub-micromolar quantities has been achieved.

The authors discussed the details of the sampling procedures and the methods used for analysis. Also, results of samples collected from human subjects were presented.

### Automated sample preparation with membrane microtitre extraction for bioanalytical mass spectrometry

**John Janiszewski, Richard Schneider, Keith Hoffmaster, Monica Swyden, Hassan Fouda**
*Central Research Division, Pfizer Inc., Groton, CT 06340, USA*
**and David Wells**
*3M Sector Research and Development, St. Paul, MN 55144, USA*

HPLC/atmospheric pressure ionization mass spectrometry is now widely recognized as an ideal, extremely sensitive, technique for the analysis of drugs in biological fluids. Its superior specificity has relaxed the demands for rigorous chromatographic resolution allowing for short runtimes (2–3 min compared with traditional techniques requiring 10–30 min per sample). As a result, sample preparation is now the rate limiting step to higher throughput. Several approaches for automated sample preparation have been reported; the most promising to date has been the utilization of solid phase extraction in a 96 well microtitre plate format. This paper described the implementation of a novel particle-loaded membrane extraction system in which the particles are uniformly distributed and held together with PTFE fibrils (90% particles w/w). This membrane system has several significant advantages over loosely packed solid phase extraction columns. The superior loadability of the thin membrane permitted large sample size and, due to its low bed volume, the analytes are eluted in a very small solvent volume (200 µl) negating the need for time consuming evaporation and concentration steps.

An instrument (Quadra 96) specially adapted to automate solid phase extraction (condition, sample load, wash and elution) in a microtitre plate format was also described. Elution is accomplished into a microtitre collection plate, which allows for direct injection from an autosampler without an additional transfer step. All factors affecting various assay performance parameters were optimized. The automated system was applied to the analysis of ziprasidone in serum. The results compare favourably with those obtained with standard solid phase and solvent extraction methods. However, significant improvements in assay throughput were realized for the current automated membrane microtitre membrane extraction method (10 min total extraction time to process 96 samples).

### Evaluation of an automated field sampler incorporating solid phase extraction disks

**Donna M. Beals, Jane P. Bibler, Dorothy A. Brooks**
*Savannah River Technology Center, Westinghouse Savannah River Co., Aiken, SC 29808, USA*
**Craig Markell, Peter Ellefson**
*3M Corp., St. Paul, MN 55144, USA*
**Ralph Setter and Robert Fiedler**
*ISCO, Inc., Lincoln, NE 68528, USA*

The Savannah River Technology Center is evaluating new field sampling methodologies to more easily determine concentrations of radionuclides in environmental (aqueous) systems. One methodology studied makes use of 3M Empore solid phase extraction (SPE) disks. The disks are composed of selective resins embedded in a Teflon support. The disks remove the ion of interest from aqueous solutions when the solution is passed through the disk. The disk can be counted directly to quantify the radioisotope of interest.

Commercial ISCO, Inc. 3700 series portable samplers were modified by engineers at ISCO, Inc. to collect aqueous samples from surface streams and process the sample through 3M Empore RAD SPE disks at the time of collection. Six modified samplers were installed at five locations around the Savannah River Site, a former nuclear production facility. (Two modified samplers were installed at one sampling location.) The samplers were programmed to collect 30 ml samples every 30 minutes. The SPE disks were changed weekly, returning the disks to the lab for quantification of the radionuclide activities. The samplers with the SPE modification were placed at routine sampling points such that the data produced by this study could be directly compared with standard laboratory based analysis results. Data collected over a four month period compared favourably with the laboratory based sample results, at significant cost and time savings.

Prior to the field study, several commercial and test Empore materials were studied for use in the modified field sampler. The extraction of four radionuclides (Tc-99, Sr-90, Cs-137 and Pu-238) by six different materials was studied. Extractions were tested from DI water, filtered and unfiltered river water and seawater. Extraction efficiency, kinetics (flow rate past the disk), capacity, and potential interferences were studied as well as quantification methods. Based on these laboratory studies, the commercial Tc RAD disk, the commercial Sr RAD disk and a test material selective for Cs were used in the field study.

### Application of automated solid-phase microextraction for the analysis of organic compounds

**Ralf Eisert and Janusz Pawliszyn**
*Department of Chemistry, University of Waterloo, Waterloo, Ontario, N2L 3G1, Canada*

The kinetics of the SPME process, which is primarily influenced by diffusion of the analytes from the aqueous sample volume through the static aqueous layer surrounding the fibre into the immobilized polymeric film, can be enhanced by intensive stirring of the solution. Thus, a very steep concentration gradient between the concentration of the analytes in the sample and the fibre increases the mass transport of the analytes into the coating. The new SPME III autosampler agitation was compared to static absorption, magnetically stirred samples, and a newly developed flow-through extraction cell. The effectiveness of the three agitation technique when using direct solid-phase microextraction from the aqueous sample was characterized. A mixture of different polar and apolar pesticides, representing a large range of K-values, had been chosen as model compounds for these investigations. The absorption–time profile was studied for all the systems evaluating the time necessary to reach the equilibrium. Two different types of vials (2 ml and 16 ml vials) had been studied characterizing the influence of the sample volume. Equilibrium is reached substantially faster for agitation techniques. The precision of the results obtained with autosampler agitation was in general better 5% RSD. Thus, the precision obtained seems to be amenable for trace analysis, taking into account that the precision of the SPME process represents the precision of the entire analysis.

### Determination of transition metals in the environmental samples by ICP-MS with on- and off-line sample preparation

**Michiko Yamanaka, Tetsushi Sakai, Hiroki Kumagai, Yosinori Inoue**
*Yokogawa Analytical Systems Inc., 2-11-13 Nakacho, Musashino-shi, Tokyo 180, Japan*
**and Elzbieta Bakowska**
*Hewlett-Packard Company, 2850 Centerville Rd., Wilmington, DE 19808, USA*

ICP-MS is a powerful tool for sensitive and selective elemental anaysis. However, some problems caused by matrix in the samples have been pointed out. Decreasing sensitivity due to a large amount of $Na^+$ and interference of polyatomic ions with $Mg^{2+}$ and $Ca^{2+}$ are typical examples. These problems obstruct the determination of transition metals (TM) and rare-earth elements (REE) in sea water or waste water. For eliminating these matrices, chelate resin, such as having iminodiacetic acid as the chelating group (IDA resin), has been widely used for sample preparation. However, since the selectivity of TM and REE against alkaline earth elements is not enough, pH adjustment and multiple eluent are required in the procedure.

In this study, the authors evaluated an alternative chelate resin having nitrilotriacetic acid as the chelating function group (NTA resin), and applied to IC-ICP-MS system for preparation of environment samples. $Na^+$, $Mg^{2+}$ and $Ca^{2+}$ were separated from TM and REE with NTA resin column at IC. Then, separated elements were introduced to ICP-MS. Using NTA resin column ($Cu^{2+}$ adsorbing capacity was 70 µmol/g), a sensitive and selective analysis of TM except $Mn^{2+}$ and REE in pseudo-sea water was accomplished by using nitric acid as the eluent. RSD ($n = 10$) of many TM were less than 3%.

### Headspace and aroma profile of sugar

**J. C. Mifsud, J. Poling and K. Weber**
*Alpha Mos, 20 Avenue Didier, Daurat, 31 400 Toulouse, France*

The smells of sugar crystals differ depending on raw materials and refining processes. An electronic nose is an instrument with a headspace sample inlet and a detector made up with arrays of gas sensors. Such an apparatus is a good compromise between subjectivity and sensory panels and the most laborious, high cost equipment based dynamic headspace GC-MS.

The volatile compounds of different types of sugar crystals have been analysed by collecting headspace on Tenax resin and thermal desorption combined with GC-MS analysis. All eight samples, originally selected by a sensory panel, were also measured by an Alpha MOS electronic nose made up with 12 gas sensors. About 30 volatile aroma compounds were tentatively identified and semi-quantitated by GC-MS. The electronic nose was capable of discriminating all the samples; samples accepted by the sensory panel were located clearly far away from rejected samples in the results. The differences in aroma profiles of GC-MS were correlated with sensory and electronic nose data.

### Quality control of packaging with the electronic nose Fox 4000

**J. Poling, Q. Lucas and K. Weber**
*Alpha Mos, 20 Avenue Didier, Daurat, 31 400 Toulouse, France*

Packaging plays a very important role in the food industry and the quality of foods. Manufacturers must package finished products with materials such as PE, PET and paper board. To meet these demands the quality of material supplied is extremely important. Since the final product quality can be influenced by quality of packaging, a company must be able to control the packaging element through suitable quality tests. Sensory panels and GC-MS are often used. An electronic nose offers the advantages of speed, no sample preparation and direct correlation with quality descriptors.

Analyses on PET pellets, paper board of various quality (different manufacturing processes, concentration of residual solvents, paper coatings, inks) have been performed with an electronic nose, FOX 4000 with 18 metal oxide sensors. The data obtained were processed with multivariate analysis (linear statistics) and neural networks (nonlinear) in order to predict the quality of unknown samples.

The FOX 4000 was shown to be successful in predicting the PE quality and is a useful tool for assessing the quality of packaging materials rapidly using small amounts of sample (a few grams).

### Automating the analytical laboratory via the chemical analysis automation paradigm

**Robert M. Hollen**
*Los Alamos National Laboratory, Los Alamos, NM 87545, USA*

To address a need for standardization within the analytical chemistry laboratories, the Chemical Analysis Automation (CAA) program within the Office of Technology Development's Robotic Technology Development Program is developing laboratory sample analysis systems that will automate both private and governmental environmental chemical laboratories. The current laboratory automation paradigm consists of islands-of-automation possessing limited capability that will not integrate into a systems architecture. Thus today the chemist must still perform many aspects of environmental sample analysis manually and work with instrumentation that generally cannot communicate physically or electronically with the outside world.

By designing and transferring to an industrial partner, SciBus Analytical Inc., of Sunnyvale, California systems based upon the Standard Analysis Method (SAM) architecture, CAA is working towards a standardized and modular approach to laboratory automation. Each SAM system will automate a specific chemical method, from the initial sample preparation through analytical analysis, and subsequently generate knowledge of the sample via knowledge-based data interpretation. The building block of a SAM is known as the Standard Laboratory Module (SLM). The SLM, being either hardware or software, automates a sub-protocol of an analysis method and can operate alone offering just sample preparation.

The CAA concept allows the chemist to assemble an automated environmental analysis system using standardized SLMs easily and without the worry of hardware or software incompatibility or the necessity of generating complicated control programs. As they are hardened for the rigours of on-site chemical analysis, these systems are being designed for use within transportable laboratories.

### Automated chemistry system control concepts and implementation

**Jerome Chen, Tracy Erkkila**
*Los Alamos National Laboratory, Los Alamos, NM 87545, USA*
**James R. Younkin and David H. Thompson**
*Oak Ridge National Laboratory, Box 2008, Bldg. 7601, Oak Ridge, TN 37831-6304, USA*

The automation goal of the Chemical Analysis Automation (CAA) program is to be able to quickly integrate or reconfigure various pieces of automated laboratory equipment produced by different vendors. At present, these pieces of equipment utilize different communications interfaces and protocols. The CAA system control software seeks to make quick integration (plug and play) possible by defining a standardized protocol through which communications between different pieces of equipment can be made uniform. The key concept of this protocol is that only equipment responses are tightly regulated. The equipment command format is regulated, but the actual command set for any specific piece of equipment is left to the vendor to define. This freedom avoids the need to 'shoehorn' functionality into a predefined command set and also avoids the need to constantly extend the command set or equipment that does not adequately fit within existing protocol definitions.

The CAA system control software operates by processing a sequenced 'script' of instrument commands that define the various operations that individual pieces of laboratory equipment must perform on a sample to complete the desired analysis. These scripts will be automatically produced by a chemist using a 'method manager' that processes the capabilities of the available equipment. The laboratory operator can then use the system control software to execute these scripts to process samples through the integrated lab bench.

### A communication infrastructure for laboratory automation

**James R. Younkin, David H. Thompson**
*Oak Ridge National Laboratory, P.O. Box 2008, Oak Ridge, TN 37831-6426, USA*
**Jerome Chen**
*Los Alamos National Laboratory, P.O. Box 1663, Los Alamos, NM 87545, USA*

The Chemical Analysis Automation (CAA) project defines the automated laboratory as a collection of standard laboratory modules (SLM) which are connected and serviced by a robot. These SLMs are designed to allow plug-and-play integration into automated systems to perform particular standard analysis methods (SAM). While the individual SLMs are autonomous in the execution of their particular chemical processing task, the SAM concept relies on a high-level task sequence controller (TSC) to route a sample through the SAM by coordinating the robotic delivery of materials requisite for SLM operations, initiating SLM operations with the chemical method dependent operating parameters, and coordinating the robotic removal of materials from the SLM when its operation has completed. Other integral components of a SAM include a human computer interface (HCI) for entering samples into a system and monitoring SLM status and sample progression and a data manager that is tasked as being the SAM's central point for data storage and exchang. A 'UNIX sockets' client/server communication infrastructure based on ethernet and TCP/IP has been developed to allow plain text communications to occur between all of these SAM constituents. This software has been used for the implementation of SAM components running under a variety of operating systems.

### A standard analysis method for the automated analysis of PCBs in soils using the chemical analysis automation paradigm

**Leon N. Klatt, James R. Younkin**
*Oak Ridge National Laboratory, P.O. Box 2008, Oak Ridge, TN 37831-6306, USA*
**Matthew Monagle, Jerome Chen and Rob Johnson**
*Los Alamos National Laboratory, P.O. Box 1663, MS J580, Los Alamos, NM 87545,USA*

The Chemicals Analysis Automation (CAA) project, sponsored by the Robotics Technology Development Program within the Office of Science and Technology, US Department of Energy is developing a standardized modular automation strategy for chemical analysis. In this automation concept, analytical chemistry is performed with modular building blocks that correspond to individual elements of the steps in the analytical process. With a standardized set of behaviours and interactions for the blocks they can be assembled in a plug-and-play manner into a complete analysis system. These building blocks, which are referred to as Standard Laboratory Modules (SLM), interface to a host control system that orchestrates the entire analytical process, from sample preparation through data interpretation. The integrated system is called a Standard Analysis Method (SAM).

A SAM for the automated determination of polychlorinated biphenyls (PCB) in soils, assembled in a mobile laboratory, has undergone extensive testing at the Oak Ridge National Laboratory. The SAM consists of the following SLMs: a four channel Soxhlet extractor, a high volume concentrator, an extract clean-up module, a gas chromatograph, a data interpretation module, a robot, and a human–computer interface. The SAM is configured to meet all the requirements specified in the US EPA SQ-846 regulations. Once the input queue is filled, samples are completed in intervals of 30 minutes. The PCB SAM will be described. Validation and performance data from the testing protocols will also be discussed.

### Chemical analysis automation standard laboratory module paradigm: the high volume concentrator module

**Michael Clark, Terry Turner, Kerry Klingler, Rod Shurtliff, Bob Kinoshita and Jeff Young**
*Idaho National Engineering Laboratory, P. O. Box 1625, Idaho Falls, ID 83415-2220, USA*

The Chemical Analysis Automation or CAA program is developing a standard for laboratory automation. The standard is built upon blocks called Standard Laboratory Modules or SLMs. An SLM is an instrument designed with standardized hardware, software, and behavioural characteristics that automatically executes a portion of an analysis method. A particular SLM might automate single aspects of sample introduction, sample preparation, or a combination of aspects. Other types of SLMs provide analysis, or the automated interpretation of the raw data. The SLM standard was developed to allow SLMs from various vendors to be grouped into Standard Analysis Methods or SAMs. A fully functioning SAM will provide automated sample analysis from sample-in to interpreted data-out. The SLM standard also provides for operation apart from a multiple SLM system. The principle tenants of the SLM interface standard as applied to equipment will be presented.

One example of an instrument utilizing the CAA SLM standard is the High Volume Concentrator or HVC. The HVC automates the Kuderna-Danish/Snyder ball column concentrator, used to concentrate trace amounts of a sample dissolved in an organic solvent. The instrument automatically reduces aliquots as large as 500 ml, to a volume of less than 10 ml during the primary process. Final volume is automatically adjusted to 10 ml through solvent addition or the volume can be reduced to 1 ml through an automated nitrogen blowdown process. The HVC performs its tasks as a stand-alone instrument, or may be combined with other SLMs into a SAM and controlled as a portion of the automated system using the CAA protocol. The HVC SLM currently is integrated into a Standard Analysis Method based on a mobile laboratory platform used to detect the presence of PCBs in soil.

The Standard Laboratory Module interface standard and the High Volume Concentrator were developed within the US Department of Energy's Robotic Technology Development Program, Chemical Analysis Automation program.

### Using a human computer interface in a standard automated laboratory

**David H. Thompson, James R. Younkin**
*Oak Ridge National Laboratory, Box 2008, Bldg, 7601, Oak Ridge, TN 37831-6426, USA*
**and Jerome Chen**
*Los Alamos National Laboratory, P.O. Box 1663, MS-J580, Los Alamos, NM 87545, USA*

The Chemical Analysis Automation (CAA) project defines the automated laboratory as a collection of Standard Laboratory Modules (SLM) which are connected and serviced by a robot. In the automated laboratory a Human Computer Interface (HCI) is required to display information from the automation controller as well as provide an interface for supervision and control of the Task Sequence Controller (TSC), all active SLMs, and samples. The CAA graphical HCI presents laboratory information to the user in a way that emphasizes samples and SLMs. The user is unaware of the TSC and all of the underlying protocol. In the analytical laboratory the chemist is primarily interested in the progress of samples. The HCI presents a list of samples including current status and a panel containing selected SLM information on the main street. Selecting a sample or SLM on the main screen gives the chemist control of sample progress or SLM state. Detailed sample information is available by double clicking a sample identifier. A sample detail window shows sample status and a history of SLM interactions and script parameters. Double clicking an SLM identifier in the main window brings up a window showing detailed information about an SLM and makes available additional control of the SLM. Other windows are used to access alarms and perform sample entry. Tool bar and menu buttons are available to control the SLMs and samples as needed. This talk presented the design and operating experience with the HCI implemented for the CAA project.

### Automated data interpretation applied to multi-compound analytes

**Leon N. Klatt and Martin A. Hunt**
*Oak Ridge National Laboratory, P.O. Box 2008, Oak Ridge, TN 37831-6306, USA*

Multi-compound analytes are encountered in numerous areas of chemical analysis, e.g. polychlorinated biphenyls (PCB) and petroleum products in environmental analysis, the measurement of gasoline octane number, bacteria identification via fatty acid patterns, and meat inspection. The instrumental data obtained in these situations generally do not contain a unique response that is simply related to the information of interest. Consequently, the analysis of the data derived from these samples usually involves the application of multivariate statistical techniques. Numerous studies in the literature have shown that different multivariate data processing methods perform differently depending on the type of multi-compound analyte sample and the type of instrumental data. The Chemical Analysis Automation (CAA) project has been developing a software Standard Laboratory Module (SLM) to provide an automated and unified capability for data processing of instrumental data from multi-compound analytes. Current work is addressing gas chromatographic data for the determination of PCBs. This SLM contains three quality assurance checks and multiple multivariate calibration and prediction algorithms. The output from each algorithm is an analyte concentration, confidence interval, and an importance parameter. The importance parameter is a scalar quantity that attempts to measure how well the unknown chromatogram is described by the standards. A fuzzy logic inference engine is used to combine these multiple outputs into a single output. This presentation described the data interpretation SLM. The performance of this SLM using data obtained from a test of a CAA Standard Analysis Method that performs automated determination of PCBs in soils was also presented.

### Marketing issues of the chemical analysis automation

**Michael A. O'Hagan**
*SciBus Analytical Inc., 764 Shasta Fir Drive, Sunnyvale, CA 94086, USA*

The Chemical Analysis Automation Program (CAA) was conceived and constructed based on the internal needs of the US Department of Energy to address a significant increase in the work load of their chemical analysis laboratories. Whether or not one believes that these increases have or will ever occur, the outcome of the CAA program to date has been the development and demonstration of an approach to automating the chemical laboratory of today with cost effective hardware and software products which streamline the laboratory's work flow.

Although the technology is developed for the environmental laboratory, there are direct applications in other industries. The introduction of products and standardization techniques are specifically targeted for the conservative nature of the chemical analysis laboratory industry. This paper discussed the characteristics of the CAA program and the way in which the needs of the general laboratory are met.

The paper explored the areas of interest to the laboratory community in a broad range of applications for the CAA hardware and software. The results of a multi-industry laboratory surve were presented which include the characteristics which are of interest such as: sample preparation and interpretation, protocols, laboratory management systems, throughput, and cost factors.

### Optimized purge and trap/GC/MS system for ultratrace analysis of volatile compounds in drinking water

**Imogene L. Chang, Matthew S. Klee and Charles Knipe**
*Hewlett-Packard Company, 2850 Centerville Road, Wilmington, DE 19808-1610, USA*

A P&T/GC/MS system was optimized to determine more than 84 EPA 524.2 targeted volatile organic compounds at ppt (parts per trillion) levels without the use of cryo-focusing. The optimized system consisted of a purge and trap concentrator and autosampler, capillary GC with a volatiles interface (VI), a wide-bore capillary column, and a new, more sensitive, bench-top quadrupole mass spectrometer with a turbo pump.

Analysis time was less than 30 minutes for quantitation of all 84 compounds. Data were acquired in full scan (35-265 amu) mode for all analytes. Without using cryo-focusing, the early eluting compounds (gases) partially co-eluted, but could be accurately quantitated using target compound analysis procedures (extracted ion determination) provided by the Chemstation software which also controls all aspects of the chromatographic system. To obtain complete separations of individual gas as preferred by some scientists, a new, efficient (use a small amount of liquid nitrogen) cryo-focusing device was used. Instrument requirements and method parameters were presented.

With or without using cryo-focusing, results for the US drinking water method (524.2) showed that this system exceeded the detection limit requirements for all 84 compounds listed. For example, all 14 aromatic compounds and 42 of 46 halogenated hydrocarbons listed in EPA Method 524.2 could be quantitated at 10 ppt or lower with a signal to noise ratio $>5$. Of the 28 remaining compounds in Method 524.2, all but four polar analytes could be quantitated at 50 ppt, the four polar compounds could be quantitated at 100 ppt.

### Automated continuous on-line measurements of VOCs in urban air in PPB and sub-PPB levels

**Felix Behm**
*Airmotec ag, Länggstr. 19, CH-8308 Illnau, Switzerland*
**and Franck Amiet**
*Chromato-Sud, 15 Route d'artiguelongue, F-33240 Saint Antoine, France*

Volatile organic carbons (VOC), CO and $NO_x$ are the precursors of the formation of ozone. Today, except for VOCs, these pollutants are surveyed continuously by commercially available monitors. AirmoVOC, an automated device based on the high resolution, high speed micro-GC from Airmotec ag, Switzerland, has been designed to fill this gap. It has all the features of a classical laboratory GC (thermal desorption unit, cryotrap, capillary columns, gradient temperature programming, column pressure control and programming, FID with parametric gas control) within a 19 in rack, it allows for automated monitoring VOCs in ambient air down to ppt levels and therefore is well introduced in European measuring networks since 1991. The instrument has a unique design to work in continuous mode due to an adsorption drum which allows sampling during chromatography. For highly volatile compounds, a cryotrap is installed, so high resolution chromatography is possible. The chromatography is made on a 10 m capillary column in 2 to 4 minutes with temperature programming and/or a column pressure program. Total cycle time is between 10 and 20 minutes.

The linearity over the range from 0.1 to 300 $\mu g/m^3$ is better than 5%, stability and reproducibility are in the range of 2%. Certification for BTX analysis was achieved according to the corresponding DIN-norm 33 963. The testing was done by RW TÜV Germany.

### Use of an atomic fluorescence gas chromatographic system to determine mercury species in ambient air samples and gas samples

**Peter B. Stockwell, Warren T. Corns and Louise Armstrong**
*PS Analytical Ltd, Arthur House, Unit 3, Crayfields Industrial Estate, Main Road, St Paul's Cray, Orpington, Kent BR5 3HP, UK*

While current legislation regarding the levels of mercury in ambient air and gas samples is based on the total mercury content, there is increasing interest on determining the mercury species present.

The PS Analytical Sir Galahad instrument has been introduced to analyse the former levels but the inherent sensitivity of the specific Atomic Fluorescence Detector recommends it as a suitable mode of detection for the latter interest. This instrument has been extended to cope with the analysis of stack gas samples. In this application it is also necessary to partially speciate the mercury levels. The configuration of a system to measure the total vapour phase mercury, preinorganic mercury and the particulate mercury will be described and data presented.

There is also evidence that the transportation of mercury in the environment is related to atmospheric cycling and therefore there is a need to determine the organomercurials in air samples. Most mercury removal systems do not tackle this problem and it is therefore vital that the distribution of mercury is determined.

Solid phase adsorbents, such as tenax, have been used to preconcentrate organomercurials in gaseous samples, followed by the subsequent desorption on to capillary GC using a novel programmable temperature vaporization injection system (PTV). This approach, which couples the separation power of the chromatographic technique with the PS Analytical Merlin Atomic Fluorescence Detector, was discussed with specific applications.

The design of a fully automated instrument which will allow continuous monitoring of urban areas, was outlined, as well as experience with related on-line applications.

### Microdevices for sample preparation based on continuously flowing streams

**Andreas Manz**
*Imperial College of Science, Technology and Medicine, Zeneca/Smith/Kline Beecham Centre for Analytical Science, London, UK*

Miniaturized analysis systems have a number of advantages, e.g. faster analysis time, better separation efficiency, lower reagent consumption. However, real samples to be analysed eventually contain particles or compounds that adsorb to walls. The micron sized, high efficiency electrophoresis system, for example, uses single channels of 5 to 50 μm diameter. In these dimensions, the surface to volume ratio is relatively large. Adsorption processes dominate, and therefore, sample preparation prior to analysis becomes a necessity.

Free-flow electrophoresis is a very attractive scheme, because it is a continuous flow method. The sample is introduced into a flat bed under laminar flow conditions. An orthogonal electric field deflects the ions depending on their electrophoretic mobility. A silicon microdevice with a total volume of 25 μl allows a continuous sample stream of 0.1 to 1 μl/min to be handled. Up to 100 V applied would give enough resolution to baseline-separate small ions of one charge difference.

Instead of using a single capillary for the electrophoretic separation, parallel capillaries can be used. Injection of an identical or different samples into capillary arrays can be done using the same procedure of voltage control as in single capillary devices. However, the geometry of the channel arrangements becomes a critical issue. A 17- and an 80-channel device have been tested. Injected samples tend to be diluted depending on channel number. Up to 46 parallel injections could be made within one load cycle of 3 seconds.

### On-line competitive immunoassay and immunobinding study using CE-LIF

**Li Tao and Robert T. Kennedy**
*Department of Chemistry, University of Florida, Gainesville, Florida 32611, USA*

The goal of this work was to develop a competitive immunoassay to monitor insulin secretion from single

Islets of Langerhans with high time resolution. In order to achieve this, an on-line competitive immunoassay for insulin was developed and applied to monitoring insulin concentration in a flowing stream. In the assay, solutions of fluorescein-labelled insulin (FITC-insulin), monoclonal anti-insulin, and sample containing insulin are pumped into a cross where they begin to mix. The mixture flows through a fused silica reactor capillary to a flow-gated interface. During transfer to the interface, a plug of the mixture is injected into a separation capillary where the bound and the free FITC-insulin are separated and detected by capillary electrophoresis with laser induced fluorescence detection. The amount of bound FITC-insulin and free FITC-insulin can be used to quantify insulin concentration. The detection limit is 270 pM. Each separation requires less than 1 s and over 12 000 consecutive assays can be acquired with no need to rinse the separation capillary. Thus, the system can be used to monitor insulin in a flowing stream for flow injection analysis or for sensor-like monitoring. Dilution and zone broadening during transfer of sample to the interface limit the response time of the on-line system to about 25 s. The authors are presently adapting the system to measure insulin secretion from islets. The fast separation and sensitive detection also allowed for direct quantification of free and bound FITC-insulin. Therefore, the binding constant between FITC-insulin and the antibody can be measured. By competitive immunoassay and theoretical simulation, the binding constants between insulin and the antibody can also be measured.

### Integrated microfabricated devices for chemical and biochemical analysis

**J. Michael Ramsey**
*Chemical & Analytical Sciences Division, Oak Ridge National Laboratory, P.O. Box 2008, Oak Ridge, TN 37831-6142, USA.*

Microfabricated devices that perform chemical analysis procedures have primarily focused on chemical separation methods. The first such device was a gas chromatograph fabricated on a silicon wafer, reported over a decade ago. This seminal work did not produce significant interest from the chemical measurements community due to low performance characteristics. More recently there has been renewed interest in microfabricated chemical instruments but with a focus on electrophoresis devices and analysis of fluids in general. In moving to the liquid state and employing electrokinetic transport phenomena, microfabricated chemical analysis devices have delivered performance features equivalent to or exceeding that of conventional laboratory instrumentation. Furthermore, these new devices show promise for integration of multiple laboratory functions that accomplish a complete assay, i.e. placing the features of an entire laboratory on a chip. Ultimately one would like to have a collection of micro-laboratory components (the analogues of resistors, transistors, etc.) that could be designed into new devices to solve specific measurement problems.

Realization of these micro-laboratory components could allow a paradigm shift for chemical and biochemical synthesis and analysis similar to that provided to electronics by the transistor and integrated circuit. Some of the components we have developed for the laboratory on a chip will be described. The use of these components in integrated devices that perform automated chemical kinetics experiments and DNA restriction fragment analyses at the subnanolitre scale was presented. Detection in nanoscale volumes places extreme demands on detection capabilities. Fluorescence detection at the single molecule level on microchips was discussed to address this problem.

### Large sample volume capillary GC and GC/MS for trace analysis

**P. Magni, F. Munari and S. Trestianu**
*CE Instruments, Strada Rivoltana, I-20090, Rodano, Milan, Italy*

Large sample volume capillary GC or GC/MS is a very powerful technique for trace analysis at ppb/ppt or even sub-ppt levels. By injecting hundreds of microlitres instead of a few microlitres, the limit of detection is lowered by two orders of magnitude and the sample preparation procedures are simplified and abbreviated by eliminating the evaporative concentration step.

The sampling system suitable for large sample volume injections are all based on sample desolvation (solvent solute separation and solvent venting) prior to the solute transfer into the analytical column. The two major injection techniques suitable for large sample volumes are:

- Cold on column coupled with desolvation precolumn and a solvent vapour exit.
- Programmed temperature vaporizer (PTV) operated in solvent split mode.

The first technique is the only one granting full sample integrity but is not suitable for polar solvents or dirty samples where the use of PTV is more indicated.

The paper described a dedicated capillary GC instrument (Ultra-Trace GC) provided with a modular multi-injector sampling system able to perform either cold on column or PTV large volume injections. Applications in environmental and food control illustrated the capabilities of the equipment for trace analysis and the advantages and limitations of the mentioned injection techniques.

### On-line analysis of vaporous hydrogen peroxide and water in isolation barriers using near-infrared spectroscopy

**Seetha Ananth and Gary Lang**
*Rosemount Analytical Inc., 1201 N. Main Street, Orrville, OH 44667, USA*

Pharmaceutical manufacturers are in need of a real-time, reliable, safe, direct method to determine hydrogen peroxide ($H_2O_2$) and moisture concentrations in isolation barriers, sterilizers and freeze dryers. Manufacturers must comply with estabished government regulations for validation and routine control of the $H_2O_2$ sterilization cycle.

Biological challenges and relative humidity (RH) sensors are currently used to determine $H_2O_2$ and moisture levels during the sterilization process. However, these techniques are qualitative and inaccurate determinations of sterility or moisture content.

The Rosemount AOTF-NIR$^{TM}$ Hydrogen Peroxide Analyser provides *in situ*, real-time, direct determination of $H_2O_2$ and moisture throughout the entire sterilization cycle. Calibration techniques, and results from actual sterilization cycles were discussed. Data were shown that illustrate the importance and benefit of using the $H_2O_2$ Analyser to monitor a sterilization cycle.

### Process supercritical fluid chromatography

**Kenneth J. Melda and Jerry M. Clemons**
*ABB Process Analytics, 843 N. Jefferson St., Lewisburg, WV 24901, USA*

Gas chromatography, although one of the most powerful separating techniques currently available, has certain limitations associated with sample volatility. While some improvements have been made with the advances in High Temperature Gas Chromatography, this extension of range puts severe strains on columns and instrument hardware. High Performance Liquid Chromatography, on the other hand, is capable of separating non-volatile mixtures since it takes advantage of the strong solvating properties of a liquid mobile phase. However, it has only been Gas Chromatography that has successfully made the transition from laboratory to process instrumentation. Due to the many detection capabilities, speed of analysis and lack of toxic solvents, Process Gas Chromatographs have gained a strong foothold in the process instrumentation market place. In the past, non-volatile sample mixtures which cannot be analysed by process gas chromatographs are taken off line and analysed in the laboratory by some alternative technique.

With the every growing tighter control on product specifications, there is becoming a greater need for the 'on-line' measurement of these low- to non-volatile samples. One chromatographic technique that shows great promise for the analysis of these samples is that using supercritical fluids. Supercritical Fluid Chromatography combines several of the favourable features of both gas chromatography and liquid chromatography, which makes it an ideal technique for analysing samples of low volatility and also a technique that is easily adaptable to 'on-line' process chromatography.

A description of a Process Supercritical Fluid Chromatograph along with several areas of applications relevant to the chemical, petrochemical and refining industries was presented.

### Automated fraction collection for packed-column SFC

**Charles R. Frey**
*Gilson, Inc., 3000 W. Beltline Highway, Middleton, WI 53562, USA*
**and Medhi Ashraf-Khorassani**
*Virginia Polytechnic Institute and State University, Department of Chemistry, Hahn Hall, Blacksburg, VA 24061, USA*

As a result of significant interest in analytical uses of supercritical fluid technology in the late 1980s, packed-column supercritical fluid chromatography (p-SFC) evolved into a viable separation tool. Due to the limitations of fixed restrictor designs in early instrumentation, pressure and flow rate could not be controlled independently and separation capabilities were limited. When the introduction of commercially available flow-independent automated pressure control in 1992, independent control of pressure and flow rate became available, significantly expanding the scope of p-SFC. The flexibility and chromatographic resolution provided by modern p-SFC technology should allow it to replace most non-aqueous normal phase HPLC methods.

The ability to collect fractions from p-SFC separations would allow the use of this technology in a preparative capacity. This could be of great value not only for efficient isolation of components, or ranges of components from a separation for further study or analysis, but also for the safety advantages of using carbon dioxide and non-toxic modifiers as a mobile phase. Regardless of these advantages, automation of the fraction collection process is required to make it a useful preparative technique.

Automation of fraction collection for p-SFC presents several challenges due to pressure requirements in the flow path and the expansion of the mobile phase as it exits the system. These challenges include the need for pressure controlling devices that introduce minimal dead volume to the flow path, collection solvents to trap the fraction components from a gaseous mobile phase as it exits the system, sealed collection vessels, immersion of the collection tube in a collection vessel solvent, and efficient cleaning of post pressure-control tubing and components. The ability to meet these needs is the key to making this a useful preparative technique.

This paper focused on the design of a p-SFC system with automated fraction collection capabilities and included data to demonstrate its use and performance.

### Microchip-based instrument systems for molecular separations and integrated biochemical processing

**Michael R. Knapp, Calvin Chow, Anne Kopf-Sill, Luc Bousse, Rob Nagle, Michael Lacy, Richard McReynolds, Theo Nikiforov, Stephen Sundberg, Claudia Cohen, Robert Dubrow, Colin Kennedy, Morten Jensen, Aileen Zhou, Sang Joeng and J. Wallace Parce**
*Caliper Technologies Corp., 3180 Porter Dr., Palo Alto, CA 94340, USA*

Feasibility studies for the development of molecular separations and microfluidic biochemical processing systems in microfabricated structures have been reported. In some successful manifestations, electrokinetic phenomena have been used as a fluidic driving force and two-dimensional interconnected channel structures have provided the necessary elements for complex injection and virtual valving strategies. However, these have remained specialty experimentation formats because the necessary instrumentation is complex and, in some cases, must be custom built.

Instrument system prototypes were introduced that will allow routine, inexpensive access to the power of microchip devices. Molecular separations data produced on these systems were presented that show how such instrumentation will provide simpler work flow and quantitative results in a fraction of the time required by conventional systems. It was shown how it will be possible to build automated experimentation systems by organizing the channel structure to recapitulate the fluidic tasks associated with sample preparation steps. The ultimate goal is to build specimen partition and fractionation elements into the microchip devices as well so that complete automation of complex biochemical analysis can be achieved. In addition to simplifying the experimentation tasks of experts, such devices will provide non-specialists with access to chemical and biochemical information that normally can only be acquired in sophisticated laboratory settings. By providing better access to such information, it is possible to change the way in which such information can be used.

### Accuracy of low-level nutrient analysis in estuarine water using flow injection analysis

**Prentiss H. Balcom, J. Richard Jadamec, Carol Anderson and Barbara L. Welsh**
*University of Connecticut, 1084 Shennecossett Road, Groton, CT 06340-6097, USA*

Study of the transport of nutrients within estuarine systems requires analytical accuracy and precision at concentrations less than 100 µg/l. While flow injection analysis (FIA) methods generate precise results, analytical system performance (accuracy and precision) is typically monitored for one analytical run only. For studies of the temporal behaviour of nutrients in the environment, the long-term performance of the analytical system is critical and needs to be characterized.

Over approximately six weeks, estuarine samples with nutrient concentrations typically less than 50 ug/l were analysed. Nitrate + nitrite, ammonium and phosphate were measured concurrently using an automated ion analyser with brackish/seawater FIA methods and photometric detection. For each analytical run, estuarine samples were analysed concurrently with calibration standards which covered the entire calibration range and were analysed in triplicate. To evaluate precision and accuracy within each analytical run, second source (quality control, QC) standards, duplicate samples and spiked samples were routinely used.

Results for calibration standards analysed as samples allowed for assessment of accuracy within an analytical run for each nutrient. Single concentration estimates of accuracy were also obtained from results for QC standards and spiked samples. The best estimates of true sample concentrations were obtained by repeat analysis on separate analytical runs (separate calibrations). Repeat analysis of calibration standards as samples on separate analytical runs allowed for estimation of the reproducibility and accuracy of estuarine sample results. An estimate of the error associated with analytical results can be made by comparison of the variability in results for calibration standard analyses between analytical runs.

The long-term accuracy and precision of FIA was examined by comparing results for calibration standards analysed as samples among analytical runs on separate analysis days. QC standard and spiked sample results were also examined from these same analysis days, and will be compared to results for calibration standards. For each nutrient, temporal patterns in precision and accuracy of analytical results were discussed. These methods for assessing accuracy of analytical results are applicable to a number of instruments used for nutrient, elemental and carbon analyses.

### Simultaneous and automatic determination of nitrogen, carbon, and sulphur in soil and sediments by dynamic flash combustion

**Liliana Krotz, Luigi Ragaglia and Franca Andreolini**
*CE Instruments, Strada Rivoltana, 20090 Rodano (MI), Italy*

The analysis of elemental constituents of soil and sediments provides useful information to identify the deficiency or excess of important nutritional elements, as well as the presence of environmental pollution. The most important parameters are nitrogen, carbon, and sulphur. Furthermore, the differentiation between inorganic and organic carbon in soil provides additional important information. Nitrogen has a great influence on soil productivity, organic carbon is linked to the metabolic process of animals and plants, while inorganic carbon plays an important role in managing the pH equilibrium of soil.

This paper dealt with the evaluation of the dynamic flash combustion method for fast, automatic, and reliable routine analysis of nitrogen and carbon in soil and sediments as a viable alternative to classic methods.

The dynamic flash combustion method is based on the instantaneous and quantitative combustion of the sample, placed in a tin container, in the presence of a fixed amount of oxygen. The combustion gases produced are then separated by a GC column and detected by a thermal conductivity detector. The total nitrogen carbon, and sulphur concentrations are then automatically calculated from the area of the relative peaks.

Differentiation between organic and inorganic carbon is achieved by first analysing an aliquot of the soil sample for total carbon content, then a second aliquot after treatment with acids for removing the inorganic carbon for obtaining the organic carbon content. The inorganic carbon content is finally calculated by difference.

### Evaluation of automated solid phase extraction for the determination of oil and grease

**Mark E. Krigbaum**
*Tekmar-Dohrmann, 7143 E. Kemper Rod., Cincinnati, OH 45249, USA*

The US Clean Air Act Amendments of 1990 require the phase out of the use of chlorofluorocarbons (CFCs). The EPA developed method 1664 to replace existing gravimetric techniques that require the use of Freon-113 as an extraction solvent for the determination of oil and grease and petroleum hydrocarbons in aqueous matrices.

Method 1664 is a performance based method that is currently written using liquid–liquid extraction techniques. However, the method does allow the use of alternative extraction and concentration techniques such as automated solid phase extraction.

This paper demonstrated a method for the extraction of oil and grease from aqueous matrices using automated solid phase extraction and 47 mm bonded silica disks. Benefits of automated solid phase extraction include reduction in solvent usage, speed of extraction, and improved data quality. The automated solid phase extraction workstation called the AutoTrace uses positive displacement pumps and an electronically controlled syringe. The author showed that the method meets the performance specifications of method 1664. Analyte recoveries, precision, accuracy, and method detection limits were evaluated.

### Quality assurance and reference material needs—a calibration standards perspective

**David T. Henderson and Kenneth J. Herwehe**
*Supelco, Inc., Supelco Park, Bellefonte, PA 16823, USA*

The US environmental industry has peaked as a growth industry and is now flat. The number of samples run each year, along with available testing revenues, has declined while quality assurance requirements are more stringent than ever. International environmental markets are growth opportunities for standards producers, however, producers find themselves in a struggle. This struggle is to improve quality systems to meet requirements of the domestic industry while concomitantly meeting the needs of the ever changing international markets.

Because quality system and product requirements differ substantially between regulating and conformity assessment groups, the costs of meeting needs across multiple markets are often beyond acceptable limits. Calibration standards manufacturers, both organic and inorganic, are therefore examining new and different ways to meet these requirements. Producers are partnering with NIST, working with purveyors of assessment and quality process registrations, and even working with competing standards producers to meet the demands of both US and international laboratories. While probably no one best approach exists, a number of quality and conformity options available within the industry were examined.

### Use of purge and trap and methylene chloride as methods for extraction of aromatic components from selected varieties of turnip greens

**G. Jones, Ola Goode Sanders**
*Dept. of Food Science and Animal Industries, Alabama A&M University, P.O. Box 264, Normal, AL 35762, USA*
**and Casey Grim**
*USDA-ARS, Southern Regional Research Center, New Orleans, LA 70179, USA*

Recently, the Cruciferous/Brassica family has been cited for its anticarcinogenic properties. Turnip greens (*Brassica rapa*), a member of the Brassica family, are commonly consumed in the southern region of the United States. Typically, turnip greens are bitter and the flavour and taste become more pronounced as the greens mature. Various methods can be utilized to isolate aromatic components from turnip greens, however, one method does not give a complete picture of the aromatic characteristics. This study focused on the use of purge and trap to extract lower molecular weight and more volatile compounds in turnip greens. Methylene chloride was used to extract bitter and less aromatic compounds in turnip greens. Three varieties (Seven Top, Purple Top and Tokyo Cross) of turnip greens harvested at 45, 60 and 75 days after planting were used as the test samples. Compounds were isolated and identified by gas chromatography and mass spectrometry, respectively. Benzene acetonitrile, benzene propane nitrile, 1H-indole-3-acetonitrile, benzene ethyl isothiocyanate (glucosinolate degradation products), aldehydes, acids and various other aromatic compounds were isolated, identified, and quantitated from each variety and maturity combination. Glucosinolate degradation products were extracted with methylene chloride; aldehydes, acids and other aromatic compounds were extracted by purge and trap. Of the four glucosinolate degradation products, only benzene propane nitrile and 1H-indole-3-acetonitrile were significantly different for variety. All glucosinolate degradation products were significantly different for maturity. The concentration of aldehydes, acids and other aromatics generally increased up to 60 days of maturity and decreased by the 75th day of maturity. Tokyo Cross had the highest concentration for most of the aldehydes, acids and other aromatics extracted by purge and trap. However, Seven Top had the highest concentration of components extracted by methylene chloride.

### A new microwave autoclave for automation of acid digestion procedures

**W. Gary Engelhart, Werner Lautenschlager and Martin Metzger**
*Milestone MLS, 7289 Garden Road #219, Riviera Beach, FL 33404, USA*

Hundreds of laboratories currently utilize microwave acid digestion systems to accelerate the preparation of samples for atomic spectroscopy. Conventional microwave digestion systems with multi-mode cavities and doors are designed for manual operation. Relatively few laboratories have invested in robotics to automate these systems, because the conversion is quite cumbersome. The greatest impediments to automation are the multi-step operations needed to individually seal, load and unload special robot-compatible vessels into the microwave cavity.

A new microwave autoclave eliminates the impediments to automating microwave acid digestion procedures. The ultraCLAVE system combines microwave heating with high-pressure vessel technology allowing analysts to perform acid decomposition procedures on samples at pressures and temperatures up to 200 bar (2900 psig) and 350°C. The system is specifically designed for semi-automated batch processing of multiple samples. Full automation is achieved by interfacing a laboratory robot arm to load/unload sample racks.

The elevated operating pressure and temperature capability of the ultraCLAVE allows acid digestion of large organic samples, (up to 50 grams), orders of magnitude larger than is possible by any other wet digestion technique.

Acid temperatures ≥ 300°C are often required to achieve complete oxidation of organic compounds with complex structures and high molecular weights. Total decomposition of samples to ionic solutions is important in ICP-MS analysis to avoid interferences and impaired instrument operation. The high operating temperatures and pressures attainable in the ultraCLAVE ensure total decomposition of complex organic samples, with no residual organic carbon content for efficient ICP-MS analysis.

Fundamental design and operating principles of the system were presented, along with representative examples of acid digestion procedures which have been conducted using the system.

### Evaluation and performance data for an automated microwave sample handling system for soils and sludges

**Graham Coe, James Allibone and Peter Walker**
*Thames Water Utilities Ltd, Spencer House Laboratory, Manor Farm Rd, Reading, Berkshire RG2 0JN, UK*

The determination of the Aqua Regia extractable metal ion concentration within soils and sludges is essential to give the limits to which sewage sludge can be disposed to land. These disposal limits are defined by law and failure to comply can lead to prosecution. The extraction process was time dependent, labour intensive and involves the manual handling of concentrated mineral acids. Through the use of microwave irradiation, sample extraction times have been greatly reduced, from hours to tens of minutes but the problems of sample handling are only now being addressed. These problems can be tackled through the use of robotic handling systems combined with microwave irradiation.

The system chosen for this study was the 'Q-PREP 5000' from Questron. It consists of a microwave digestion system with on-line sample handling and cooling. A range of parameters was selected for evaluation with response measured by the efficiency of extraction from a range of soil and sludge types including standard reference materials. These parameters were time of microwave irradiation; sample mass and particle size; extraction medium; day-to-day reproducibility and comparison to both bomb microwave and heating block extraction. Additionally the use of the on board predigestion step was investigated.

Analysis of the data produced showed that time of extraction is the limiting factor with a cut off period beyond which no further improvement in extraction efficiency is observed, this being 300 seconds. Sample particle size was found to be critical in the evaluation when comparing like with like, in both sample type and extraction method. Data for the other parameters presented, show that a good correlation exists between extraction methods and that reproducible results are obtainable for the robotic system compared to the other two methods. With the addition of a pre-digestion step, difficult sample matrices can be extracted including sewage sludge and vegetable matter.

### Automated perchloric/hydrofluoric acid digestion of ore samples, tailings, and NIST sediment for metals analysis

**Edward J. Hinderberger, Kevin P. Kelly**
*OI Analytical, Sample Preparation Products Group, 555 Vandiver Drive, Columbia, MO 65202, USA*
**and Jean-Luc Lenoir**
*Prolabo, 54 rue Roger Salengro, F 94126 Fontenay-sous-Bois Cedex, France*

Samples of copper ore, ore tailings and sediment in open Teflon vessels were digested using multiple sequential additions of perchloric, nitric, hydrochloric, and hydrofluoric acids with a 200 W microwave system integrated with automated robotics for unattended sample transfer and reagent additions. Fumes from the acid digestion were evacuated through a scrubbing system. The samples were also prepared by a conventional procedure using Teflon beakers with acid digestion on a hot plate.

Analytical results from the analysis of samples prepared by conventional hot plate digestion were comparable to those from samples processed using the automated sample preparation system. The NIST Buffalo River sediment sample was used as a reference material. Throughput for the automated microwave digestion system was 10 to 12 samples per day; however, the entire procedure is unattended, and labour requirements for the automated system were approximately four to six times less than those for the manual digestion method. Use of automation also eliminated the potential for contamination consequent to the repeated opening and closing of vessels required during the manual procedure.

### Automated HPLC for toxicological and therapeutic drugs monitoring

**Steven H. Wong, Mark McGuire and Rebecca D. Cannon**
*Department of Pathology, Medical College of Wisconsin, Milwaukee, WI 53226, USA*

Laboratory automation offers cost-effective testings for the current managed care delivery system. In the clinical laboratories, high throughput analysers, coupled with immunodiagnostic reagents, have been successfully used for drugs/metabolites screening or quantitation. For forensic urine drug testing, automated urine sample preparation with commercially available workstations prior to GC/MS analysis may be performed for the five drug groups required by SAMHSA. For pharmaceutical laboratories, automation by coupling column switching and/or robotic stations with chromatographs have afforded dedicated automated analysis of investigational new drugs as part of the research and development processes. Currently, commercial systems are available for applications in clinical and pharmaceutical laboratories. This presentation reviewed three levels of automation associated with chromatographic analysis of drugs/ metabolites, followed by selected clinical analyses using commercially available systems, Level III HPLC automation.

Three levels of automated chromatographic analysis are proposed to enhance protocol selection. Level I automation would include automated sample preparation modules, column switching, and automated HPLC with data reduction capability. Cited in the literature, this approach is often characterized as 'totally automated' analysis. Level II automation includes added robotics and/or column switching with limited data reduction. Level III would include commercially available systems with flexibility of column switching, robotic liquid or solid phase extraction and microdialysis, and chemometrics for data reduction and/or drug/metabolite identification.

For toxicological drug identification, the commercially available Level III HPLC, REMEDi™-HS, using chemometrics, is capable of automated preparation of urine/serum samples by column switching, followed by multimodal analysis and chemometrics-enhanced drugs/ metabolites identification. Currently, REMEDi™-HS is capable of identifying 760 therapeutic and illicit drugs/ metabolites. Of these, 70 have been approved by the FDA for *in vitro* clinical diagnostic testing. These would include commonly used therapeutic drugs such as antidepressants, cardiac drugs, opiates, over-the-counter medications such as antihistamines, and illicit drugs such as cocaine metabolites and amphetamines. Another commercially available, Level III system—PrepStation™ and HPLC was used to analyse felbamate and tricyclic antidepressants, and to check light-sensitive nifedipine dosage preparation. Felbamate, an approved but recently discontinued antiepileptic, was analysed by an automated liquid–liquid extraction protocol. Serum containing felbamate was mixed with acetonitrile/internal standard for protein precipitation. After axial centrifugation and standing, supernatant was transferred and injected into the HPLC for sequential analysis and data reduction. An automated HPLC protocol using PreStation™ was developed for solid-phase extraction of tricyclic antidepressants. To the conditioned DAU extraction cartridges, serum mixed with an internal standard was introduced for extraction and later, elution with methylene chloride/isopropanol/ammonia. Finally, light-sensitive nifedipine dosage analysis was performed by automated dilution protocol using PrepStation™, followed by HPLC analysis to confirm dosage concentration. Amber vials were used for this protocol to minimize light exposure. The above examples demonstrated the clinical as well as unique applications of automated HPLC for toxicological and therapeutic drug monitoring.

### The fate of mercury in sample preparation

**Peter J. Walter and H. M. 'Skip' Kingston**
*Duquesne University, Department of Chemistry and Biochemistry, Pittsburgh, PA 15282-1503, USA*

Mercury is a toxic element in many chemical forms whose analysis is hampered by its intrinsic mobility and volatility. There is a potential for loss of mercury during every stage of sample preparation and analysis. Sample preparation can lose mercury during drying, digestion, and reactions of the mercury with vessel walls. Analyses of mercury are also hampered by mercury reacting or sticking to components of the analysis system. Two approaches to solving these problems were evaluated: identification of sources of mercury loss during every stage of sample preparation; and an alternative analysis of mercury through direct analysis of solid materials, requiring little or no sample preparation.

Traditional sample preparation steps were evaluated for mercury quantitation. Drying of the sample in an oven, vacuum drying, and other techniques rely on different mechanisms for drying which show varying degrees of mercury loss. Typically, acid digestion of a sample in a closed vessel retains the mercury, but many analysis techniques require matrix conversion after digestion. This conversion may inadvertently unstabilize the mercury ions, leading to elemental loss. Alteratively, by using an automated mercury analyser, the sample can be directly analysed without sample preparation. This technique is capable of on-line decomposition of the sample in an oxygen furnace while collecting the mercury as an amalgam. Subsequent release of the mercury into a detector leads to total mercury analysis at the nano-gram level. This approach has the potential to be a field technique, as well as being compatible for direct and decomposed samples.

### Modelling and optimization for multiphase high-speed GC separations

**Richard Sacks, Heather Smith and Mark Nowak**
*Department of Chemistry, University of Michigan, An Arbor, MI 48109, USA*

Emerging technologies have made high-speed gas chromatography (HSGC) both practical and commercially available. Much of this work has involved the development of efficient inlet systems which can generate very narrow vapour plugs. This is necessary to take full advantage of the resolving power of short capillary columns operated at high carrier gas flow rates. However, the use of short columns significantly reduces the available peak capacity. This has limited the application of HSGC to relatively simple mixtures. The use of tunable columns, where the polarity and selectivity can be tuned for a specified set of target compounds, can result in much greater efficiency in the utilization of available peak capacity and dramatic reductions in analysis time. Tunability is achieved by the use of a serially-linked (tandem) ensemble of two or more columns of different selectivities with adjustable carrier gas pressures at the junction points between the columns. Changing these pressures changes the relative contributions that columns make to the overall separation selectivity. Optimization strategies are based on the determination of the tuning pressures which will give the greatest resolution of the most difficult to separate component pair. Optimization strategies were described for ensembles containing two and three columns and for the case where isothermal column temperature as well as junction pressures are optimized.

A vector model of multiphase separations was also presented. In this model, retention factors for all mixture components for the different column types are plotted in an orthogonal coordinate system where each axis corresponds to a different column type. Every mixture component occupies a single point in this multidimensional retention space. An overall retention axis then is constructed from the origin, and the angle of the axis with respect to the orthogonal co-ordinate system is determined by the fractional contribution that each column type makes to the overall ensemble selectivity. It was shown that orthogonal projections of the points representing the mixture components onto the overall retention axis directly shows the relative locations of all peaks and their relative separations in the resulting chromatogram. Further, a set of separation vectors was defined which connect all possible pairs of points representing the mixture components. This set of vectors completely defines the multiphase separation, and results in greatly simplified optimization procedures.

### Cyanide speciation utilizing UV irradiation and distillation within a single automated module

**Nabih P. Kelada**
*ASTM Cyanide Task Group Chairman, 609 Midway Park, Glen Ellyn, IL 60137, USA*

Few automated methods for cyanides have been previously developed. Like the manual methods, they are subjected to wide variability and several interferences, particularly the thiocyanate. The automated method given in this presentation was completely free of thiocyanate interference. In addition to the common automated components (sampler, pump, colorimeter, recorders and data systems), the system utilizes two different automated units, one for ultraviolet (UV) irradiation, and the other unit for continuous thin film distillation. For total cyanide, the UV irradiation is performed in an alkaline medium and a Pyrex sample coil (UV range about 300 nm). The strong cyanide complexes (Fe and Co) are effectively broken down, but not thiocyanate. Thus, the colorimetric determination gives the proper total cyanide value. Whereas using a quartz irradiation coil (UV full range) and acidic medium includes all the thiocyanate in the total measurement. Thiocyanate is determined by difference of the two previous measurements. On the other hand, running the system without UV irradiation gives only the dissociable cyanide (free simple cyanides and the weak complexes).

These Kelada automated methods for cyanide speciation have been adopted by the American Society for Testing and Materials, designation ASTM, D-4374 (94) *Automated Methods for Total Cyanide, Dissociable Cyanide and Thiocyanate.* The corresponding round robin study for performance evaluation gave successful results, about 5% coefficient of variation, and 90–100% spike recovery for most sample matrixes. The method, as found by the participants, is applicable to water, wastewater (effluent, raw sewage and sludge) and industrial discharges.

In the present work, automated advances have been developed. Both UV irradiation and distillation steps are made simultaneously by one newly developed automated module. The different factors and controls of UV irradiation and distillation, such as heat, time, filters, pH, coil size and diameter have been optimized within this module. This UV-distillation apparatus can accommodate three channels simultaneously for analysis of total cyanide, dissociable cyanide and thiocyanate, and can be utilized with either continuous segmented flow or unsegmented flow injection systems. A detailed description of this apparatus and the performance characteristics of the automated system was presented, and improved treatments of some interferences (nitrite, aldehydes, oxidants, and thiocyanate) with cyanide analysis were discussed.

### A flow-through extraction cell for automated SPME

**Ralf Risert and Janusz Pawliszyn**
*Department of Chemistry, University of Waterloo, Waterloo, Ontario, N2L 3G1, Canada*

A newly developed system for automated analysis of thermally labile organic compounds in aqueous samples based on solid-phase microextraction coupled to high-performance liquid chromatography was discussed. A new SPME–HPLC interface was built based on capillary SPME modified for the extraction of aqueous samples (1.4 ml volume) containing the target analytes, i.e., phenylurea pesticides. The aqueous sample is aspired and dispensed (25 μl) from the aqueous sample vial at a flow rate of 63 μl into a piece of capillary column, i.e. Omegawax 250, where the analytes are absorbed. The aspire/dispense step is repeated several times. After the extraction the extracted compounds were desorbed by aspiring methanol into the capillary and transferred into a usual HPLC injection loop. The entire SPME handling was controlled by the autosampler. The extraction cell process is characterized by a good precision below 6% RSD ($N = 10$) for the investigated compounds. The selectivity can be easily modified by changing the coating of the capillary for trapping the analytes. The results indicate the effectiveness of the extraction process when using this new flow-through cell technology.

The direct on-line coupling with HPLC using the extraction cell (capillary) either by absorption and desorption steps makes the technique capable for easy autosampler application. The SPME technique is now extended to the fully automated analysis of thermally labile target analytes.

### Continuous and discontinuous treatment of solid samples prior to immunoassay of pesticide metabolites

**M. D. Luque de Castro, M. M. Jiménez-Carmona, A. Velasco-Arjona**
*Department of Analytical Chemistry, Faculty of Sciences, University of Córdoba, E-14004 Córdoba, Spain*
**J. J. Manclús and A. Montoya**
*Integrated Lab. Bioengineering, Polytechnic University, Valencia, Spain*

The persistence of pesticides and their metabolites in soils and vegetables is one of the environmental target problems at present. Selective or specific methods for identification and/or quantitation of new compounds are being developed in order to fulfil the requirements in this field. Immunoassay has proved to be one of the fastest and most selective ways for analysis of pesticides and their degradation products. Using specific monoclonal antibodies, a competitive enzyme immunoassay for the analysis of 3,5,6-trichloro-2pyridinol (TCP, the main metabolite of chlorpyrifos) has been developed. The application of this assay for analysis of solid samples after appropriate sample pretreatment procedures was proposed.

Two automated modes for treatment of soils and vegetables prior to immunoassay have been developed.

*Robotic sample treatment,* which involves weighting of the samples, leaching for 2 h, evaporation, filtration. The filtrate is diluted as required and subjected to competitive immunoassay using a commercial autoanalyser. In this way the overall procedure is developed without human intervention.

*Continuous leaching treatment using, (1) supercritical* $CO_2$ *and, (2) subcritical water at 250°C.*

1. The extraction of the analyte by SC-carbon dioxide requires the presence of a modifier (1 ml of methanol) and an ion-pair agent (0.5 ml of 0.1 M ammonium camphorsulphonate in methanol). Complete recovery is achieved after 15 min (flow-rate 1 ml/min, temperature 40°C, pressure 383 bar and 0.95 g/ml).

2. The use of water at 250°C without (flow-rate 4 ml/min, pressure 200 bar) is sufficient for complete extraction of the analyte in 15 min.

The determination range of TCP is between 5 ng/ml and 5 μg/ml with excellent precision of both the pretreatment and quantification steps.

### Improvements to in-line UV digestion methods for total nitrogen and total phosphorus in waters using flow injection analysis

**David Diamond and Ninglan Liao**
*Zellweger Analytics, Lachat Instruments Division, Applications Department, 6645 W. Mill Rd., Milwaukee, WI 53218, USA*

The usual methods for phosphorus and nitrogen in waters are those based on USEPA methods 364.4, for total phosphorus and 351.2 for total Kjeldahl nitrogen. These methods are extremely labour intensive, with digestions of up to 2.5 hours. To address the need for faster, more efficient digestion in-line UV digestion Flow Injection Analysis (FIA) methods for the determination of total phosphorus and total nitrogen were developed. Digestion was performed using a flow-through digestion module, incorporating a UV light source and aluminium block heater.

For total phosphorus, polyphosphates are hydrolysed with acid and heat, and organophosphates are digested by UV-catalysed persulphate oxidation. The orthophosphate formed is reacted with molybdate, and reduced to phosphomolybdenum blue by ascorbic acid. For total nitrogen, nitrogen-containing compounds are oxidized to nitrate using UV-catalysed persulphate oxidation. Nitrate is then determined using the Griess reaction which uses cadmium reduction to nitrite followed by the formation of an azo dye.

The methods gave recovery of > 95% for all the organic and inorganic phosphorus and nitrogen compounds tested. The methods were validated with influent and effluent samples from wastewater treatment plants as well as with seawater. Results include comparison of in-line methods to the conventional batch digestion methods. Both methods have a sample throughput of greater than 25 samples/hour and a range of 0–10 mg P or N/L. The methods have RSDs of 0·5–1·5% and method detection limits of 0·005 mg P/L and 0·2 mg/N/L, respectively.

### Automated analysis of oil and grease samples with SPE membranes

**Michael C. Janes**
*Horizon Technology, 8 Commerce Dr., Atkinson, NH 03811, USA*

The increased costs and the environmental hazards of freon-113 as an extracting solvent has placed much emphasis on oil & grease method 1664. This new extraction method uses n-hexane as the extracting solvent which replaces freon-113. This performance based method allows the use of Solid Phase Extraction (SPE) as an extraction alternative, offering many advantages over traditional Liquid Liquid Extraction (LLE).

SPE reduces the extracting solvent by a third and emulsions are eliminated, resulting in higher recoveries. Automating SPE allows the laboratory to reduce labour significantly and increase productivity. Reproducible data and cost reductions make automation a requirement to stay competitive and remain on the cutting edge.

This paper discussed the quality of data produced using the SPE-DEX® 4750. Recovery data were presented comparing SPE, which more closely matches freon-113, to traditional LLE using n-hexane. Typical oil & grease samples have a high Total Suspended Solids (TSS) value requiring several filtration techniques. The physical interaction between the filtration media and the oil and grease were reviewed.

### On-line integration of DNA sequencing sample preparation to capillary gel electrophoresis

**HongDong Tan and Edward S. Yeung**
*Ames Laboratory-USDOE and Department of Chemistry, Iowa State University, Ames, IA 50011, USA*

Sample preparation and purification can be time-consuming, labour-intensive, and cost-effective processes in DNA sequencing. To maintain the speed, reliability and sensitivity which capillary gel electrophoresis provides to gene typing, DNA mapping and DNA sequencing applications compared with slab gel electrophoresis, one obvious approach is on-line coupling of an automated DNA sequencing reaction machine to capillary gel electrophoresis. This study showed a microfluidic interface integrating the fluorescently dye-labelled terminator cycle sequencing strategy to PEO gel-filled capillary. The cycle sequencing reactions were performed at a 250 μm-i.d. fused silica capillary in a hot air thermal cycler. After on-line purification and transfer of the reaction products, the on-line injection purification and transfer of the reaction products, the on-line injection to PEO gel-filled capillary is accomplished at a cross junction housed in the high temperature chamber. The high temperature at the purification column and injection cross prevents the renaturation of DNA fragments during on-line transfer.

### Automated micro sample handling in capillary LC

**Johannes P. C. Vissers, Jean-Pierre Chervet and Jean-Pierre Salzmann**
*LC Packings USA, 80 Carolina Street, San Francisco, CA 94103, USA*

Bioanalytical and pharmaceutical compounds often require sample pretreatment, e.g. derivatization in the case of amino acid analysis or endoproteinase digestion for peptide mapping, before they can be separated and identified by reversed phase liquid chromatography. As sample sizes tend to get smaller in volume and lower in concentration, automated on-line microsampling and handling—clean-up, preconcentration, derivatization, etc.—gets to be very important in order to prevent the loss of a valuable and/or minute sample. The limited sample availability and low sample concentration further require sensitive analysing techniques for obtaining sufficient information to characterize the samples.

This paper presented an automated microsampling workstation that is especially designed for on-line sample clean-up and automated sample injection in micro and capillary liquid chromatography. The untreated sample is injected into the system. The instrument offers different pipetting and dispensing routines that allow for the mixing of liquids and derivatization of compounds on the submicrolitre level. Among many typical applications are the development of sample handling techniques for the production of combinatorial libraries from single beads or *in situ* on-tray digestion of proteins. The column switching capabilities of the instrument are used for automated sample clean-up by use of different chromatographic steps, sample extraction and preconcentration of trace amounts of analyte from complex matrixes, detergent removal from protein/peptide samples, followed by separation by micro or capillary LC.

### Simulated distillation of petroleum products with metal capillary columns

**Allen K. Vickers and Dean Rood**
*J&W Scientific, Blue Ravine Road, Folsom, CA 95630, USA*

Simulated distillation is the chromatographic analysis whereby the boiling point range of a hydrocarbon mixture is correlated to that of the chromatographic retention times of n-alkanes. The information gained from this analysis is used in a variety of ways from raw materials quality control to verifying final product specifications.

Fused silica capillary columns have been routinely employed for the simulated distillation since 1985. Compared to the packed column counterparts these columns are more dependable from the standpoints of reproducibility, column bleed and overall system lifetime.

This paper examined an improved stainless steel tubing that overcomes one of the weaknesses of the fused silica capillary column. That weakness is the upper temperature limit of the columns which become brittle upon repeated exposure to temperature about 370°C. Much of the enhanced performance is derived from the deactivation process imposed on the inside of the stainless steel tubing. As a result, GC analysis temperatures of up to 450°C are routinely employed with the thin (stationary) film columns. The metal tube simulated distillation column shows exceptionally low bleed characteristics that allow boiling point distribution range of initial boiling points of 70°C to final boiling points near 1090°C. Other thicker film columns made with this tubing show similar improvements for the application of ASTM Method 2887 involving 'lower' temperature simulated distillation analysis.

### Determination of arsenic and selenium in water using a piezoelectric crystal detector with palladium coating

**Y. S. Fung and Charles C. W. Wong**
*Department of Chemistry, The University of Hong Kong, Pokfulam Road, Hong Kong*

The technique of hydride generation/atomic absorption spectrometry provides a very sensitive method for the determination of hydride-forming elements such as As, Sb and Se in water. The disadvantage of such a technique is the use of delicate equipment and high cost using AAS, which makes the technique not suitable for use in field monitoring. Thus, a piezoelectric (P/Z) detection method was developed which is capable of providing a portable detector for direct monitoring of these elements in water samples.

Sodium borohydride solution was used as the reductant for the generation of the volatile hydrides which were found to adsorb on the surface of the piezoelectric quartz crystal electroplated with different metals—Ni, Cu, Pd, Ag, Au and Pt. Palladium was found to be the best metal for adsorption of the hydrides. In the continuous flow system, the response of the detector was found to increase with the weight of the hydrides adsorbed and hence related to the amount of As and Se in the sample solution. Under optimized conditions, the working ranges were 50–150 ppb/10–50 ppb and the detection limits were 50 ppb/10 ppb for As/Se, respectively. The results obtained are within the guideline values specified in WHO for drinking water quality. Other hydride-forming elements such as Sb, Bi and Te were also found to adsorb on the palladium-coated crystal with different sensitivities. Most common metals, except mercury, show no interference. Using GF-AAS as the reference method for comparison, the mean recovery of As was found to be 96.3% using the P/Z method as compared to 99.2% using the GF-AAS method.

### Monitoring groundwater beneath an abandoned firefighter training facility with solid phase microextraction, gas chromatography, and gas chromatography/mass spectrometry

**H. T. Mayfield, M. V. Henley**
*Armstrong Laboratory/EQL, 139 Barnes Drive, Suite 2, Tyndall AFB, FL 32403-5323, USA*
**and T. J. Shelley**
*Applied Research Associates, AL/EQL, 139 Barnes Drive, Bldg. 1117, Tyndall AFB, FL 32403, USA*

Firefighter areas in the USA are facilities built on air force bases to train firefighters in special techniques needed to rescue people and extinguish fires in crashed airplanes. Firefighter training areas usually include an aircraft mock-up and a surrounding pond upon which fuel can be spread and ignited for realistic exercises. Although newer facilities provide liners for groundwater protection and/or use clean fuels such as liquefied petroleum gas, a number of inactive facilities were operated without liners.

Solid Phase Microextraction (SPME) coupled with Gas Chromatography (GC) and Gas Chromatography/Mass Spectrometry (GC/MS) has been used to monitor groundwater near an inactive, unlined firefighter training area. Operations at this site left a quantity of free petroleum product which can be recovered from selected monitoring wells, as well as contaminated groundwater containing water-soluble jet fuel components and other contaminants. SPME/GC/MS has been used to identify contaminants present in the groundwater and SPME/GC has been applied to the periodic monitoring of the contaminants. Headspace SPME proved useful in the examination of the contaminated groundwater samples which contained a wide range of contaminant concentrations.

### Oil and grease analysis comparing solid phase extraction and liquid–liquid extraction with hexane and Freon™

**Richard M. Pieper, Li Song, Eric Wisted and Craig Markell**
*3M New Products Department 3M Center, St. Paul, MN 55144, USA*

The change from Freon™ based liquid–liquid extraction (LLE) for oil and grease analysis to the new EPA Method 1664 analysis based on a hexane LLE, has prompted consideration of solid phase extraction (SPE) as a possible sample preparation procedure instead of the liquid–liquid analysis. Early work presented by 3M showed that in comparison to the EPA Method 413.1, Freon™ LLE, SPE disks are an acceptable alternative.

The present study was conducted specifically to compare the hexane LLE with the SPE disks. Sixteen waste streams were sampled.

Samples were taken from factories representing industries such as animal fats, food preparations, fabricated metals, aluminium die casting, laundries, sewerage systems, snack foods, rubber products, cosmetics, and candy products. Four replicate samples were analysed by hexane LLE and four replicates were analysed by SPE. Referee samples were analysed by Freon™ LLE.

Results of the 16 different waste streams yielded the following information:

- One sample could not be analysed by hexane liquid–liquid analysis.
- Of the remaining 15 samples, 10 samples analysed by hexane liquid–liquid and solid phase extraction were in close agreement.
- Of the five samples not showing favourable agreement between hexane LLE and SPE all five samples were analysed by Freon™ LLE which compared favourably to the SPE.

### Analysis of organic pollutants in water with fully automated on-line solid phase extraction and HPLC/DAD

**Li Wang, Hong Wang, John E. George III, Gary K. Ward and Jerry J. Thoma**
*Environmental Health Laboratories, 110 S. Hill Street, South Bend, IN 46617, USA*

High performance liquid chromatography (HPLC) with diode-array detection (DAD) is a very promising technique for the analysis of large groups of polar and thermolabile pollutants. In this study, HPLC/DAD was used to analyse water samples for a large number of semi-volatile organic compounds many of which are currently analysed by other methods (GC and GC/MS). Incorporated with the HPLC/DAD is a fully automated on-line solid-phase extraction with a PROSPEKT module (Varian). On-line solid-phase extraction is very cost effective as it eliminates the need for manual sample preparation and reduces the required sample volumes. Sensitivity and reproducibility are improved since extraction is conducted automatically and consistently for each sample and the entire extract is eluted into the HPLC system.

In this method, a small volume of sample was pumped onto a conditioned PLRP-s cartridge by using a PROSPEKT automated solid phase extraction unit. The extracted analytes on the cartridge were then eluted directly into an HPLC system, separated by a C-18 column with gradient pump and detected by DAD.

Most of the compounds studies were extracted and separated efficiently and analysed at low levels (ppt) in drinking water. This method was compared with other EPA 500 series methods. Results of the validation study were presented and will include dynamic linear range, method detection limit, reproducibility, matrix effects, separation and extraction efficiency.

### Open focused microwave sample preparation for total and mercury species determination in environmental samples

**Mao Tseng, Alberto de Diego, Agnes Woller, Agnes Cosnier, Vincent Schmitt and Olivier F. X. Donard,**
*Laboratoire de Chimie Bio-Inorganique et Environnement, Hélioparc, 64000 Pau, France*

The determination of mercury in environmental samples has seen considerable development in the past 20 years.

However, sample preparations are always complex. The authors have used an open focused microwave system for the rapid determination of total mercury in sediment. The same sample preparation strategy was slightly modified and allowed to obtain good results on certified reference material for mercury species (sediments and biological tissues).

For the total mercury determination, the sample digestion procedure was adapted to ICP/MS determination. Certified sediment samples were digested with a two step procedure using a $HNO_3$ and $H_2O_2$ mixture under mild conditions. A complexing agent EDTA and Triton X-100 were added to minimize memory effects in the ICP/MS FIA system.

For the mercury species determination in biological tissues, an alkaline digestion procedure was added under mild operating conditions. Determination of the mercury species was performed using cryofocusing techniques/atomic absorption spectrometry after hydride generation. A slightly modified digestion procedure (with 2 M $HNO_3$) was used for sediment analysis to obtain the certified values in reference materials. All procedures are simple and performed within less than 3 minutes with no loss of mercury. The paper described the different methods used and discussed their limitations.

### Speciation of mercury in lakewater with an IC/ICPMS method

**Mark Heintz and Brian Buckley**
*Environmental and Occupational Health Sciences Institute, Rutgers University, Piscataway, New Jersey 08855, USA*

Mercury contamination is found throughout the eastern US because of the volatility of mercury metal. Mercury in the environment is readily converted to organic forms via several biological mediated pathways. The concentration of total mercury in lakewater is low when compared to a matrix such as sediment, and the analysis of water samples would be expected to be of a low priority. Unfortunately biomagnification of organomercury species in the water by many biological systems can be a factor $10^6$ or even higher.

Total mercury levels in lakes with high levels of mercury in the fish are typically 10–100 ppt. Methods for measuring total mercury levels and for the separation and quantification of inorganic mercury and methylmercury levels in lakewater have been developed, and were discussed in this presentation. The total mercury levels are measured with an ICPMS system optimized for mercury analysis. These numbers are verified by the addition of a $^{200}Hg^{2+}$ internal standard. The greatest difficulty in this measurement is the determination of the mercury background for the solution. Several methods for elimination of the mercury background caused by desorption for the ICP spray chamber were also discussed.

Measurement of the different species in the water were conducted with a sensitive IC/ICPMS method developed by the authors' research group. Currently detection limits for methylmercury and the inorganic mercury species are of the order of a single ppt. These detection limits are sufficient in most cases for the measurement of the inorganic mercury species, which comprises the bulk of the mercury in the water. Ambient methylmercury levels are only a small fraction of the total mercury concentration, therefore a preconcentration step is necessary before IC/ICPMS analysis. Preconcentration is accomplished by forming a sulphur containing organic complex of the methylmercury and trapping it on a C18 column. Because of the complexity of the lakewater samples the recovery yield of the methylmercury is sample dependent. Consequently, an isotopically labelled methylmercury internal standard is added to the lakewater before exraction to compensate for the variable extraction yields. The results of these species-specific measurements were also discussed in this presentation.

### Evaluation of an on-line versus an off-line SFE-GC/MS method for the analysis of SVOCs from Tenax-filled cartridges

**James R. Bowyer**
*ManTech Environmental Technology, Inc. 2 Triangle Dr., Research Triangle Park, NC 27709, USA*
**Joachim D. Pleil**
*US Environmental Protection Agency, Research Triangle Park, NC 27711, USA*

Supercritical fluid extraction (SFE) has become an important alternative to Soxhlet extraction of analytes from environmental samples primarily because of the shorter turnaround time for analysis, reduced solvent exposure and disposal problems, and comparable extraction efficiencies. SFE has been shown to be comparable to Soxhlet extraction for extracting analytes from a variety of substrates.

The objective of this study was to evaluate and compare two SFE methods of introducing extracts from 1-ml Tenax-filled cartridges into a bench-top GC/MS system. This paper presented results of the comparison of a prototype on-line SFE-GC/MS interface and an off-line SFE GC/MS system. The evaluation was based on the efficiency of extracting semivolatile organic compounds (SVOCs) from non-sorbent material and Tenax-TA. The samples were prepared by spiking the cartridges with standard solutions of selected SVOC compounds and extracting them by using the two SFE methods. Extracts were then analysed by GC/MS, and the recovery and extraction efficiencies determined. Results indicated that the methods were equally good at extracting some compounds from the cartridges, but the on-line method had better recovery from the more volatile SVOCs. Other on-line method advantages over the off-line method includes less sample handling and indications of lower limits of detection.

The information in this document has been funded wholly or in part by the US Environmental Protection Agency under Contract 68-D5-0049 to ManTech Environmental Technology, Inc. Mention of trade names does not constitute endorsement or recommendation for use.

### Use of an on-line diluter to extend the analytical working range and improve productivity in AAS

**Glen Carnrick and Sarah Howden**
*Perkin-Elmer Corp., 761 Main Ave, Norwalk 06097, USA*
**and Jörn Baasner**
*Bodenseewerk Perkin-Elmer GmbH, Postfach 10 17 61, Überlingen, Germany D-88647*

A disadvantage of atomic absorption in comparison to optical emission or mass spectrometry techniques is the limited analytical working range. Since the graphite furnace is a discrete sampling technique, samples that are beyond the analytical working range are typically 'diluted' by using a smaller sample aliquot. When the flame is used as the atomizer source, this 'dilution' option is not possible, thus samples that are beyond the analytical working range must be diluted off-line or rerun again using a calibration curve established using a less sensitive wavelength. Both of these options are a hindrance to laboratory productivity.

One approach to dilutions for flame atomic absorption is to use a peristaltic pump to reduce the rate at which the sample is delivered to the flame. This approach, however, can lead to accuracy and precision errors due to the required flow rates for the on-line dilution process. Additionally with peristaltic pumps, the constant expansion and contraction of the pump tubing results in stretching of the tubing and a change in flow rate over time.

An alternative approach to on-line dilution utilizing stepper driven syringe-type pumps was discussed. Unlike peristaltic pumps, syringe-type pumps are capable of maintaining precise, accurate sample delivery over a long period of time. Performance and applicability of this approach to improving flame atomic absorption productivity was discussed.

### Determination of total mercury in soil samples using an iodine-based extractant

**Norma L. Ayala, Tye Ed Barbar**
*Department of Chemistry, Sam Houston State University, Huntsville, Texas, 77341-2117, USA*
**Ralph R. Turner**
*Environmental Sciences Division, Oak Ridge National Laboratory, P.O. Box 2008, M.S. 6036, Oak Ridge, Tennessee 37831-6036, USA*
**and D. F. Foust**
*Environmental Laboratory, General Electric Company, P.O. Box 8, Schenectady, New York, 12301-0008, USA*

The ideal field method for the determination of total mercury contamination would be simple and safe. Current standard methods for the determination of mercury cannot be easily adapted to field methods because they require the use of reagents that are difficult or too hazardous to handle in the field. For the vast majority of soil samples collected for analysis, the only information that is critical is the determination of whether or not mercury contamination is above the regulatory limits. For soil samples, the regulatory limits are set at a few hundred parts per million. The field analytical technique must be capable of determining when these regulatory limits are exceeded and provide Yes/No (Contaminated/ Not Contaminated) type of information.

